# Genome-protective topoisomerase 2a-dependent G2 arrest requires p53 in hTERT-positive cancer cells

**DOI:** 10.1158/0008-5472.CAN-21-1785

**Published:** 2022-05-03

**Authors:** Nicola Lockwood, Silvia Martini, Ainara Lopez-Pardo, Katharina Deiss, Hendrika A. Segeren, Robert K. Semple, Ian Collins, Dimitra Repana, Mathias Cobbaut, Tanya Soliman, Francesca Ciccarelli, Peter J. Parker

**Affiliations:** 1 Protein Phosphorylation Laboratory, The Francis Crick Institute, 1 Midland Road, London NW1 1AT, UK; 3 Centre for Cardiovascular Science, University of Edinburgh, Edinburgh, UK; 4 Division of Cancer Therapeutics, The Institute of Cancer Research, London, SW7 3RP, UK; 5 Cancer Systems Biology Laboratory, The Francis Crick Institute, London NW1 1AT, UK; 6 School of Cancer and Pharmaceutical Sciences King's College London, New Hunt's House, Guy's Campus, London, SE1 1UL, UK; 7 Barts Cancer Institute, Queen Mary University London, Charterhouse Square, London EC1M 6BE, UK

**Keywords:** p53, Topoisomerase2a, cancer, hTERT, PKCε

## Abstract

Topoisomerase 2a (Topo2a)-dependent G2 arrest engenders faithful segregation of sister chromatids, yet in certain tumor cell lines where this arrest is dysfunctional, a PKCε-dependent failsafe pathway can be triggered. Here we elaborate on recent advances in understanding the underlying mechanisms associated with this G2 arrest by determining that p53-p21 signaling is essential for efficient arrest in cell lines, in patient-derived cells, and in colorectal cancer organoids. Regulation of this p53 axis required the SMC5/6 complex, which is distinct from the p53 pathways observed in the DNA damage response. Topo2a inhibition specifically during S phase did not trigger G2 arrest despite affecting completion of DNA replication. Moreover, in cancer cells reliant upon the alternative lengthening of telomeres (ALT) mechanism, a distinct form of Topo2a-dependent, p53-independent G2 arrest was found to be mediated by BLM and Chk1. Importantly, the previously described PKCε-dependent mitotic failsafe was engaged in hTERT-positive cells when Topo2a-dependent G2 arrest was dysfunctional and where p53 was absent, but not in cells dependent on the ALT mechanism. In PKCε knockout mice, p53 deletion elicited tumors were less aggressive than in PKCε-replete animals and exhibited a distinct pattern of chromosomal rearrangements. This evidence suggests the potential of exploiting synthetic lethality in arrest-defective hTERT-positive tumors through PKCε-directed therapeutic intervention.

## Introduction

The Topo2a-dependent G2 arrest is a poorly understood control mechanism that is defective in numerous tumour-derived cell lines (1,2). This arrest mechanism is triggered by Topo2 inhibitors such as ICRF193 and if compromised triggers emergent dependence on PKCε failsafe pathways, wherein loss or inhibition of PKCε drives division failure (3).

The limited insight into this control mechanism is in part attributed to the multiple context-dependent responses that ICRF193 has been shown to elicit. Through its characteristic strand passage reaction, Topo2a is required for several biological processes, including the resolution of topological problems associated with DNA replication (4–7) and in the maintenance of telomeres in cells dependent upon the alternative lengthening of telomeres (ALT) pathway (8–12). Furthermore, ICRF193 has historically been used to drive genotoxicity and a G2 DNA damage response (DDR) (13–15), although observations in normal, diploid human cell lines show no overt DNA damage associated with its use (1,2).

The distinctive cellular behaviours consequent to Topo2a ICRF193-inhibition indicate that the elicited 'stress' responses vary, reflecting either a common Topo2a associated control pathway interpreted distinctly under different conditions or the triggering of specific cellular responses indicative of distinctive molecular contexts. A recent screen for Topo2a-dependent G2 arrest regulators provides a framework to assess this functionally (2). In this screen, p21 was identified as a non-redundant component in the ICRF193-induced G2 arrest in diploid RPE1 cells, while not impacting a bleomycin-induced arrest. The p53-p21 mediated arrest has been well characterised in the DDR pathway and prior studies focused on DNA damage involved the use of ICRF193 to identify p53 as a player in this response (13). It is unclear whether these findings are reflective of a DDR or a parallel pathway with some shared components, and if the latter, whether the mechanism is conserved in all Topo2a engagement contexts. Here we provide compelling evidence that there are context-dependent, distinctive parallel pathways that differentially engage the PKCε protective pathway when compromised.

## Materials and Methods

### Reagents, Biological and Computational Resources

For a full list of reagents and computational resources see Supplementary Table S1. For a full list of cell lines see Supplementary Table S2. All cell lines have been authenticated by STR profiling and mycoplasma screened by a PCR-based approach by Cell Services at The Francis Crick Institute.

### Cell Synchronisation

For studies of S phase a single thymidine block was performed. Cells were cultured for 16 h in growth medium supplemented with 2.5 mM thymidine and subsequently washed and released into growth medium containing 1xEmbryoMax nucleosides. For all other synchrony experiments a double thymidine block was performed as described in (2).

### Drug treatments

Unless otherwise indicated, the following drug concentrations were used in all experiments; ICRF193 3 μM, Bleomycin 10 μM, Nocodazole 1 μM, Chk1 inhibitor CCT244747 1 μM, Chk2 inhibitor CCT2415331 1 μM, ATM inhibitor 10 μM, ATR inhibitor 10 μM, Camptothecin 1 μM, Hydroxyurea 4 mM, 0.5 μM BLU577, and 1 μM BIM-1.

### EdU Click-iT Proliferation Assay

Cells were incubated with 10 μM EdU for 30 mins, then fixed and permeabilized with PHEM buffer (60 mmol/L PIPES pH6.8, 25 mmol/L HEPES pH7.4, 10 mmol/L EGTA pH8, 4 mmol/L MgSO_4_, 4% paraformaldeyhyde, and 0.1% Triton X-100) for 20 minutes. Cells were then incubated with Click-iT reaction mix containing 1 mM CuSO_4_, 1mM Azide Alexa-Fluor 488 or 546 and 100 mM ascorbic acid in PBS.

### Flow Cytometry

Cells were fixed in ice-cold 70% ethanol for at least 30 minutes and then permeabilised with 0.1% Triton X-100. Cell staining and subsequent analysis was performed using anti-MPM2-Cy5 and propidium iodide as described in (2).

### Immunoblotting and Immunoprecipitation

Whole cell lysates were obtained by sonication of cells in ice-cold RIPA-buffer (2) or 9 M Urea (9 M Urea, 150 mM 2-mercaptoethanol, 50 mM TRIS-Cl pH7.5) supplemented with cOmplete EDTA-free Protease Inhibitor Cocktail, PhosSTOP and 1 mM PMSF. Lysates were run with 1X NuPAGE LDS-sample buffer (Invitrogen).

Insoluble extracts were obtained by lysis on ice for 10 mins in 5x pellet volumes of 1% Triton X-100 buffer (1% Triton X-100, 150 mM NaCl, 50 mM Tris pH7.4 and supplemented with cOmplete EDTA-free Protease Inhibitor Cocktail (Roche)). Lysates were subject to centrifugation (13,000 x *g*, 4°C, 10 min) and insoluble pellets resuspended in 2X NuPAGE LDS-sample buffer (Invitrogen).

Immunoprecipitation was performed as described in (2), with the addition of PhosSTOP in the RIPA buffer and incubating the supernatant with anti-phospho p53 (Ser15) antibodies.

Proteins were separated by SDS-PAGE and transferred to either PVDF or Nitrocellulose membranes. Membranes were blocked and incubated with primary antibody at 4°C overnight in either 5% fat-free milk dissolved in PBS + 0.1% Tween 20 (PBST) or with 2.5% BSA in PBST. Antibodies were detected using HRP-conjugated secondary anti-rabbit and anti-mouse antibodies and Luminata HRP substrate or SuperSignal West Dura Extended Duration Substrate. A representative image of at least three experiments is shown. Band densitometry was performed using FIJI software and normalised to the appropriate control, as described in figure legends.

### Immunofluorescence Imaging and Analysis

Immunofluorescence experiments, G2 determination and co-localisation analysis were performed as described in (2). Primary antibodies used and the addition of DAPI or Phalloidin are indicated in figure legends. PCNA in synchronised cells was quantified as previously described (16).

Immunofluorescence signal intensity for p53, phospho-Ser15 or γH2AX was quantified using a custom-built script and the commercial software package MATLAB. Maximum intensity projections were made of serial z-stack images spanning the entire nucleus. Individual nuclei were identified through DAPI segmentation, background removed via thresholding and the fluorescence signal per nucleus was calculated. Normalisation to the untreated control was used to account for biological replicates. At least 30 cells were analysed per experiment and the mean and S.E.M. of at least four experiments was quantified.

Binucleate determination was performed blinded to treatments, manually counting >100 cells per condition. For the decoded data, the mean and S.E.M. of three experiments was quantified

### Mitotic Trap Assay

Unless otherwise indicated, cells were treated with 3 μM ICRF193 or 10 μM Bleomycin in combination with 1 μM nocodazole for 18 h. Data are normalised to the nocodazole alone condition.

### Organoid Establishment, Sequencing and Culture

Colorectal cancer organoids were established from fresh colorectal cancer tissues (Human Tissue Act License number 12121, REC 12-EE-0493 and 18-EE-0025). The establishment and propagation of the organoids was based on previously published protocols (17). Somatic mutations for the p53 mutant sample were determined by the South London Medicine Centre and genomic DNA for the p53 WT organoid was sequenced by the Advanced Sequencing Facility at the Francis Crick Institute.

Colorectal organoids (see Table S2) were seeded by resuspension in basement membrane extract (BME) with media (70/30 Ratio). Plates were left for 30 minutes at 37°C and 5% CO_2_ for BME to solidify before the addition of media supplemented with of 10 μM Rock inhibitor Y-27632. For passaging, organoids were dissociated by resuspension in TrypLE Express for 15 minutes at 37°C. Dissociation was stopped by addition of 5% FBS, organoids were further disrupted by pipetting multiple times and then filtered through a 70 μM cell strainer before replating.

### siRNA transfection

Cells were reverse transfected with a final concentration of 20 nM of the indicated siRNAs using Lullaby according to the manufacturer's guidelines. Where multiple transfections were performed the concentration of each siRNA was 20 nM and single transfections were complemented with non-targeting control siRNA. 72 hours of siRNA-mediated knockdown was used for all experiments. All siRNAs are specified in Table S1.

### Tumour-prone Mouse Model and analysis

Studies in animals were approved by the Animal Ethics Committee of the Francis Crick Institute and the UK Home Office. p53/PKCε mice were generated in the Biological Research Facility at The Francis Crick Institute crossing C57BL/6J Trp53^tm1Brd^ with C57BL/6J Prkce^tm1Bsca^ following ARRIVE guidelines. Mice were culled at the onset of tumour-associated symptoms, such as breathing difficulties.

DNA was isolated from frozen or from formaldehyde fixed tumour tissues using the AllPrep DNA/RNA/Protein Mini Kit (Qiagen) and AllPrep DNA/RNA FFP Kit (Qiagen), respectively. 1x Low-pass genome sequencing was performed by the Advance Sequencing Facility of The Francis Crick Institute and copy number estimation was performed using the QDNASeq package (18).

For histopathology, samples were processed and analysed in the Experimental Histopathology facility of The Francis Crick Institute. Tumour samples were fixed in 10% NBF for 24 hours and changed into 70% Ethanol. Samples were embedded in FFPE, tissues were sectioned and stained with H&E, Caspase 3 (rabbit anti-Caspase 3, R&D, AF835) and Ki-67 (rabbit ant-KI67, Abcam, ab15580). Stained tissues were examined, and mitotic index (number of mitoses per ten x400 fields), KI-67 index and Caspase-positive cells were quantified (percentage positive per x40 field, counted in regions with highest positive density).

### Statistical Analyses

A one-way analysis of variance (ANOVA) or two-way ANOVA was used for experiments with one or two independent variables, respectively, both with multiple comparison adjustment. Students t-tests were used to compare two sets of independent data. Where data has been normalised, a one-sample T-test has been used to compare to the normalised control set at 1. The statistical test for each experiment is indicated in the figure legends. Prism software was used for all calculations and the level of statistical significance is represented as follows: not significant (ns)=*P*>0.05, *=*P*≤0.05, **=*P*≤0.01, ***=*P*≤0.001 and ****=*P*≤0.0001. All statistical tests were two-sided. Sample size for each experiment is displayed in the legend.

## Results

### p21 and p53 are non-redundant regulators of the Topo2a-dependent G2 arrest

**Figure 1:**
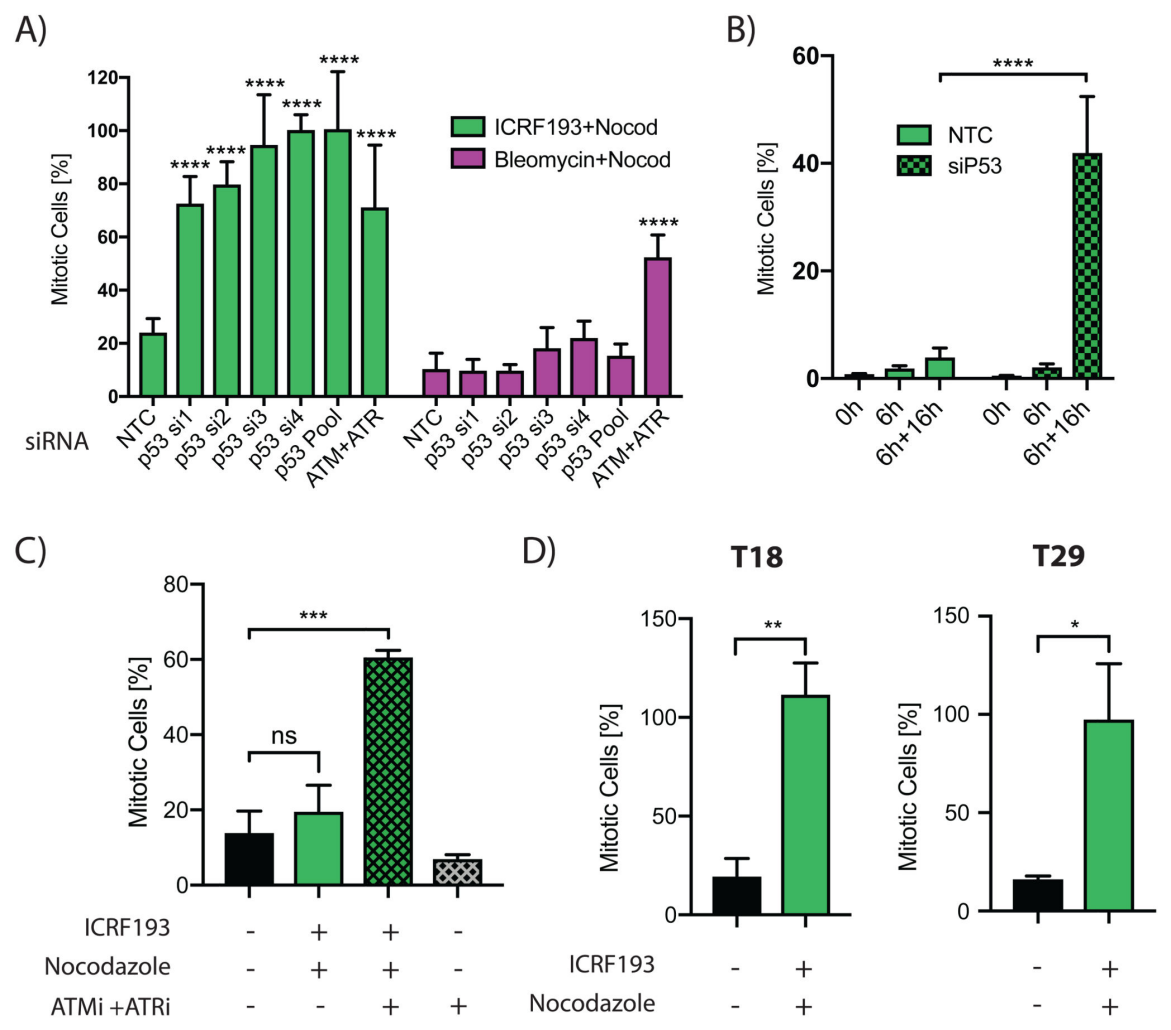
A,B) n=3; analysis by two-way ANOVA. A) Mitotic trap assay of RPE1 cells transfected with individual siRNAs, pools or a non-targeting control (NTC) or positive control (siATM+siATR). Data represented as mean±SD of a representative experiment with 6 technical replicates. B) RPE1 cells were transfected with NTC or p53 OnTargetPlus siRNA, synchronised with a double thymidine block (0h), released for 6 h (6h) to reach G2 and then treated with ICRF193 + Nocodazole for a further 16 h (6h+16h). Data were normalised to 6h+16h nocodazole alone condition and show mean±SEM. C,D) Patient-derived colorectal organoids (described in Supplementary Table S2) that were either p53 wild-type (C) or mutant (D) were treated for 24 h with ICRF193, Nocodazole or ATM inhibitor with ATR inhibitor (ATMi+ATRi). Data are normalised to Nocodazole alone and show mean±SEM, n=3 (in (C) ATMi+ATRi n=2). Analysis (C) a one-way ANOVA and (D) a t-test.

A previous RNAi screen showed knockdown of p21 abrogated the ICRF193-induced Topo2a-dependent G2 arrest and not the Bleomycin-induced DDR in RPE1 cells (2), a finding substantiated independently using a mitotic trap assay (Supplementary Fig. S1A and B). Interestingly, the p21 inducer p53 was not identified in the RNAi screen (2). This was a false negative, as deconvolution of the p53 siRNA pool used in the screen and new siRNA oligonucleotides showed that p53 loss abrogated the ICRF193-induced G2 arrest but not the DDR (Fig. 1A; Supplementary Fig. S1C-E). Importantly, the p53 requirement for efficient ICRF193-induced arrest manifests in G2 (Fig. 1B; Supplementary Fig. S1F).

To assess the penetrance of dependence, we performed a mitotic trap assay on an array of cancer cell lines with different p53 states (19). We found that cells were unable to efficiently arrest in response to ICRF193 when there was no wild-type p53 present, furthermore the G2 arrest was lost completely when cells were p53 null. Importantly, the Bleomycin-induced G2 arrest was completely functional in all cell lines tested (Table 1; Supplementary Fig. S1G).

To validate these observations in a tumour model, we performed a mitotic trap assay in patient-derived colorectal organoids that were either p53 WT or p53 mutant (Supplementary Table S2). The p53 WT organoid arrested in G2 in response to ICRF193 in an ATM- and ATR-dependent manner (Fig. 1C). In agreement with the cell line data, the p53 mutant organoids did not efficiently arrest in response to ICRF193 treatment (Fig. 1D).

### Distinct regulation of p53 in the Topo2a-dependent G2 arrest by the SMC5/6 complex

**Figure 2:**
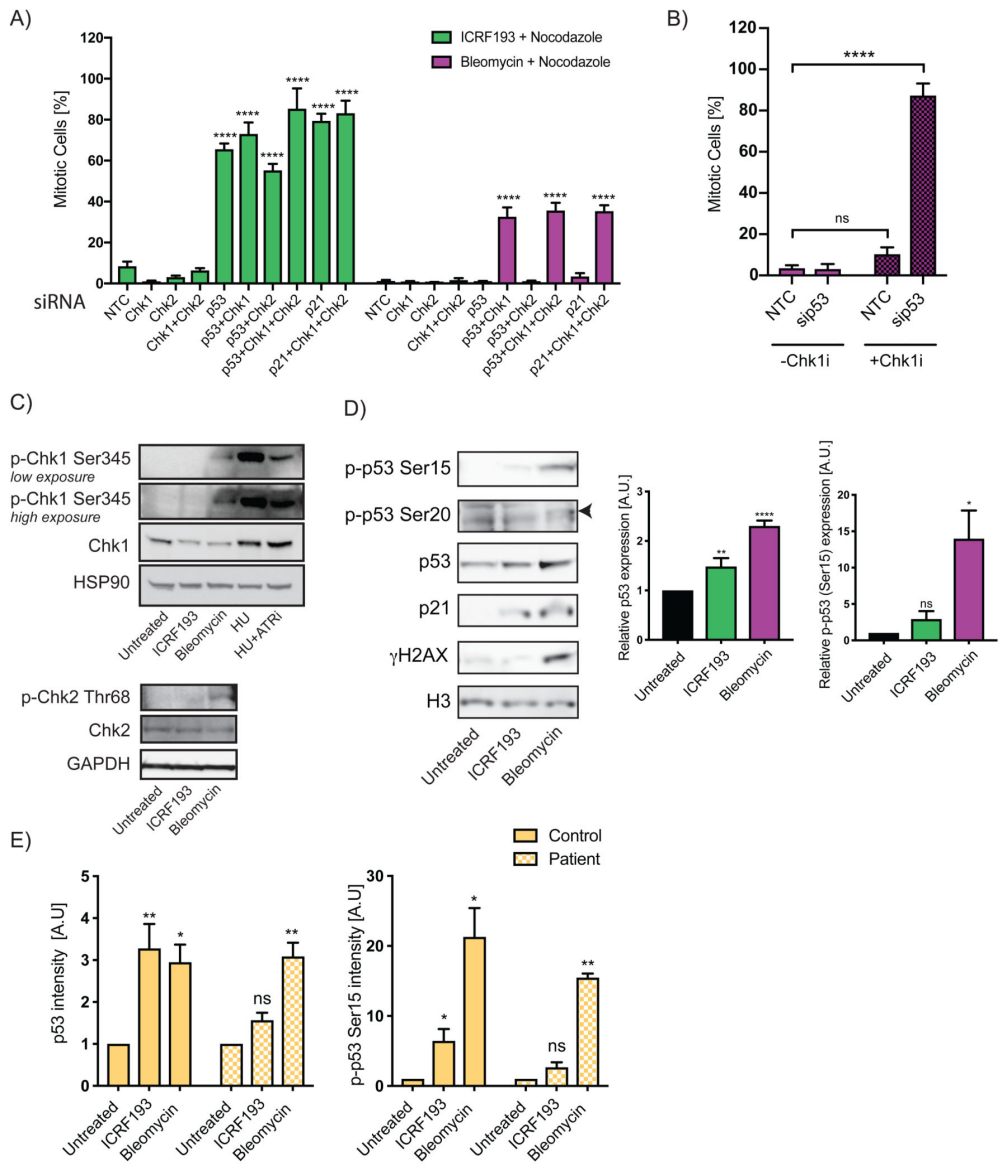
A,B) n=3; analysis by two-way ANOVA. A) Mitotic trap assay of RPE1 cells transfected with the indicated siRNAs. Data are represented as mean±SD of a representative experiment with 6 technical replicates. B) RPE1 cells were transfected with non-targeting control (NTC) or p53 OnTargetPlus siRNA (sip53), synchronised with a double thymidine block, released for 6 h (G2 phase) and treated with Bleomycin and Nocodazole and with Chk1 inhibitor CCT244747 (Chk1i) where indicated for a further 16h. Data were normalised to 16 h of Nocodazole alone and show mean±SEM. C,D) Western blots of RPE1 nuclear extracts after 18 h treatment with ICRF193, Bleomycin, 2.5 mM hydroxyurea (HU) or ATR inhibitor (ATRi). Representative experiments of n=3 are shown. Arrowhead indicates band of interest. Graphs denote the mean±SD, n=3. p53 levels were normalised to GAPDH expression and phospho-p53 at Ser15 (p-p53 Ser15) expression was normalised to p53 expression. Normalisation to the untreated control accounted for biological replicates. Statistical analysis was performed using a one-sample t-test. E) Quantification of immunofluorescent expression per nucleus of p53 or phospho-p53 at Ser15 (p-p53 Ser15) of control or patient fibroblasts when treated with ICRF193 or Bleomycin. Patient fibroblasts were NSE2 mutant (p.Ser116Leufs*18/p.Ala234Glufs*4). Data are represented as mean±SEM, n=4, where at least 30 cells are quantified/condition. Data was normalised to the untreated control and analysed by a one-sample t-test.

Despite being dispensable for implementing the DDR G2 arrest, both p53 and p21 are upregulated in response to damage (20,21). Previous reports demonstrate that Chk1/Chk2 alone can initiate a G2 arrest when cells are subjected to DNA damage and may work redundantly with p53 (15), hence we anticipated differential engagement of Chk1 and Chk2. We confirmed that loss or inhibition (22,23) of Chk1 and Chk2, either independently or in combination, was not sufficient to bypass either the ICRF193- or Bleomycin-induced G2 arrest in normal p53 replete RPE1 cells (Fig. 2A; Supplementary Fig. S2A-C). However, we observe a loss in the G2 arrest triggered by DNA damage when either p53 or p21 are knocked down in combination with Chk1 (Fig. 2A; Supplementary Fig. S2D). This redundancy is a G2 behaviour, as only the combined treatment (p53 knockdown and Chk1 inhibition) bypassed the bleomycin-induced arrest in synchronised cells (Fig. 2B; Supplementary Fig. S2E). In agreement with this, we detected phosphorylation of Chk1 and Chk2 at residues responsible for their activation (Chk1-Ser345; Chk2-Thr68 (24,25)) when cells were treated with Bleomycin, but not with ICRF193 (Fig. 2C).

In response to DNA damage, p53 expression is regulated by Ser15 and Ser20 phosphorylation by ATM/ATR and Chk2 respectively (21,26-29). These phosphorylation events alongside increased expression of p53 and p21 were observed with Bleomycin-induced DNA damage, with γH2AX signal verifying DNA damage (Fig. 2D; Supplementary Fig. S2F-H). ICRF193-treatment resulted in an increase of p53 and p21 expression and detectable Ser15 phosphorylation that is absent in untreated cultures (Fig. 2D; Supplementary Fig. S2F-H). However, no Ser20 phosphorylation was observed (Fig 2D; Supplementary Fig. S2H). Importantly, there was no DNA damage in these normal diploid cell lines with ICRF193-treatment, consistent with the conclusion that this is a distinct pathway (Fig 2D; Supplementary Fig. S2H).

The non-requirement of Chk1/Chk2 may reflect an unusual single arm of the G2 DDR pathway working through p53 regulation in response to ICRF193-inhibited Topo2a. To address this, we used fibroblast cells derived from a patient with severely reduced levels of NSE2 (a subunit of the SMC5/6 complex) resulting from a rare germline mutation (30), that are compromised in their Topo2a-dependent G2 arrest (2). Using control NSE2 WT fibroblast cells, we observe an increase in p53 expression and Ser15 phosphorylation with ICRF193-treatment, but this is not observed in the NSE2 mutant patient-derived cells (Fig. 2E; Supplementary Fig. S2I and J). Importantly the Bleomycin-induced DDR is functional within these NSE2 mutant patient cells (Fig. 2E; Supplementary Fig. S2I-L). To provide further evidence on the distinctiveness of upstream triggers, we used siRNA targeting p53, Topo2a and SMC6 in combination with Chk1 and Chk2 and performed mitotic trap assays in RPE1 cells. SMC6 and Topo2a do not act in concert with Chk1 and Chk2 in DDR (Supplementary Fig. S2M).

The evidence indicates the pathway engaged to implement the ICRF193-induced G2 arrest is a non-redundant Topo2a, SMC5/6 complex, ATM/ATR, p53, p21 regulatory cascade. Moreover, the requirements of this pathway are either not essential (Topo2a, SMC5/6 complex) or act redundantly (p53, p21) with the DDR requirement for Chk1 in G2.

### Topo2a inhibition delays S-phase progression generating unresolved replication intermediates that persist in mitosis

**Figure 3:**
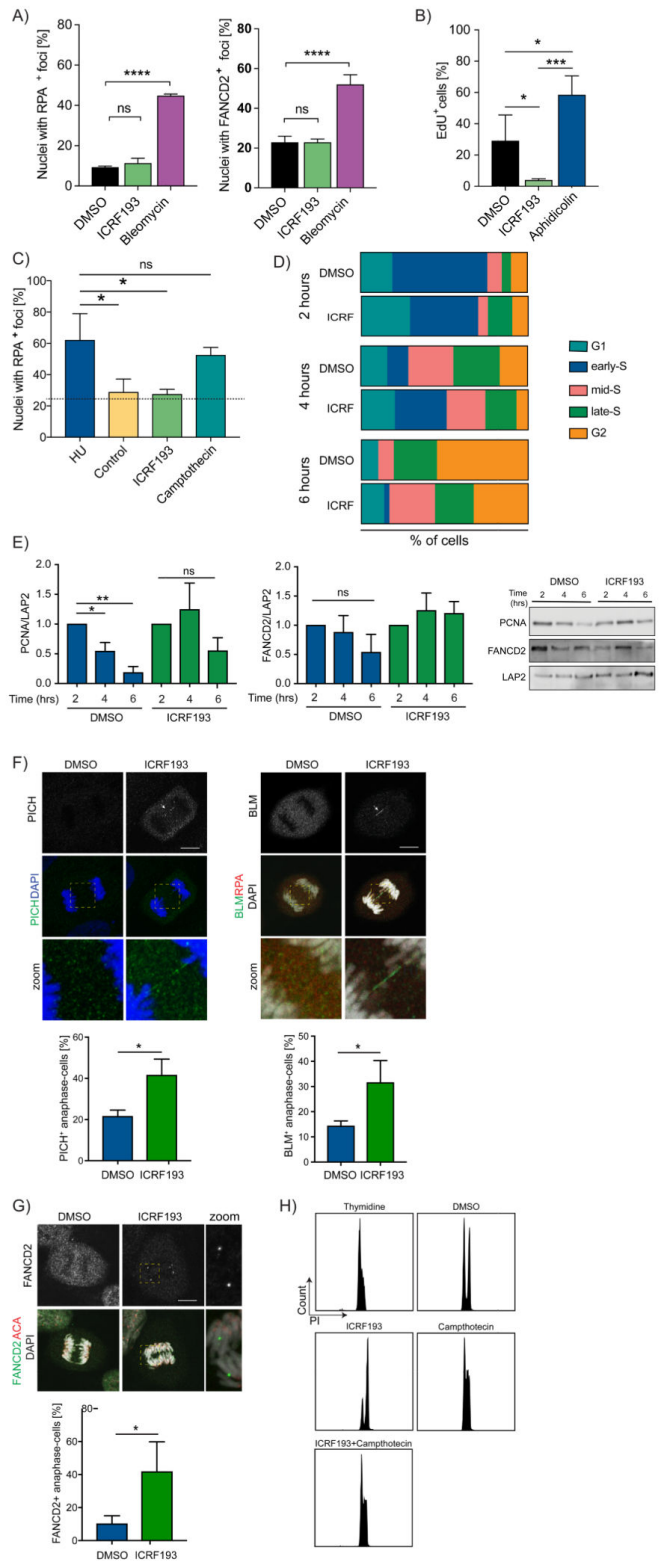
A-H) Data are represented as mean±SEM, n=3. A) Graphs represent the percentage of RPE1 nuclei with RPA2/32 (RPA) (left) or FANCD2 (right) foci. Cells were ICRF193- or Bleomycin-treated for 18 h. At least 50 cells/condition/experiment quantified. Analysis by two-way ANOVA. B) Quantification of EdU-positive RPE1 cells DMSO-, ICRF193- or 0.5 μM Aphidicolin-treated for 16 h. At least N=100 cells/condition/experiment have been quantified. Analysis performed by one-way ANOVA. C) RPE1 synchronised in G1/S, released with Hydroxyurea (HU) for 45 minutes and incubated with HU, ICRF193 or Camptothecin. Graphs show the quantification of RPA-positive foci/nuclei for each treatment. Dotted lines indicate the percentage of nuclei of asynchronous cells containing RPA foci; analysis was by one-way ANOVA. D) PCNA nuclear distribution (cell percentage) from G1 to G2. RPE1 cells synchronised in G1/S, released with DMSO or ICRF193 for 2, 4 or 6 hours. At least N=100 cells/condition quantified. E) Western blot of insoluble extracts from cells synchronised in G1/S and released with DMSO or ICRF193 for 2, 4 or 6 hours. Graphs showing PCNA (right) and FANCD2 (left) normalised to LAP2 expression. Analysis performed by t-test. F) Representative images of RPE1 cells synchronised in G1/S and ICRF193-treated for 4 hours. Left: PICH (green) and Right: RPA (red) and BLM (green). DNA detected with DAPI. Arrows: ultra-fine bridges; Zoom: PICH- or BLM-bridges. Scale bar, 10 μm. Quantification of PICH-positive (left) and BLM-positive (right) ultra-fine bridges in DMSO- or ICRF193-treated cells. 20 anaphase cells/condition/experiment have been scored; analysis by t-test. G) FANCD2 (green) and centromeres (ACA, red) staining in RPE1 cells synchronised in G1/S, 4 hours ICRF193-treated and monitored into mitosis. Zoom, FANCD2 sister foci. Scale bar, 10 μm. 20 anaphase cells/condition/experiment have been scored. Statistical analysis by t-test. H) DNA content of RPE1 cells synchronised in G1/S and released with ICRF193 or Camptothecin for 6 hours.

It is possible that the Topo2a-dependent G2 arrest arises from a G1-like DDR trigger prompted during S-phase, where ICRF193 impacts replication fork progression causing S-phase delay (6). Hence we evaluated ICRF193-induced S-phase effects on the G2 arrest. Upon ICRF193-treatment, there was no evidence of single stranded DNA (ssDNA) or stalled replication forks in asynchronous RPE1 cells, RPA2/RPA32 (RPA) and FANCD2 staining respectively, contrary to the effect of Bleomycin (Fig. 3A; Supplementary Fig. S3A). Additionally, the low levels of EdU detected in ICRF193-treated cells reveal the lack of ongoing replication when Topo2a activity is compromised, unlike following replication stress induced by Aphidicolin (Fig. 3B; Supplementary Fig. S3B).

To exclude replication as the trigger of the Topo2a-dependent G2 arrest, we sought to determine whether recovery from replication stress is a Topo2a dependent process. We synchronised RPE1 cells in G1/S and treated with hydroxyurea (HU) to induce replicative stress. After HU release, cells were either fixed as a control, or allowed to recover from the induced stress for 2 hours in DMSO, ICRF193 or the Topo1 inhibitor Camptothecin. RPA quantification revealed that Topo1, and not Topo2a, is involved in the replication stress recovery pathway in normal diploid cells (Fig. 3C). Similarly, FANCD2 staining confirmed this finding, although 2 hours release from HU was not sufficient to fully recover from the replication stress induced (Supplementary Fig. S3C).

To further investigate the potential involvement of Topo2a in DNA replication in normal diploid cells, we synchronised RPE1 cells in G1/S and tracked S-phase progression under ICRF193 treatment. Monitoring the PCNA nuclear pattern established by the replication timing programme (16) (Supplementary Fig. S3D) we observed a delay in S-phase progression in ICRF193-treated cells after 4 hours, which became more evident at 6 hours (Fig. 3D). We confirmed this at the protein level, observing significantly higher expression of PCNA and FANCD2 in nuclear extracts obtained at 4 and 6 hours post thymidine release (Fig. 3E).

Replication fork progression can be problematic in some specific DNA regions such as common fragile sites (CFSs), potentially leading to under-replicated DNA that engenders ultrafine bridges (UFBs) connecting sister chromatids in anaphase-telophase (31). When this occurs, the mitotic DNA synthesis pathway (MiDAS) is required to resolve incomplete replication during mitosis. We monitored anaphase cells derived from a synchronised RPE1 population incubated with ICRF193 only during DNA replication and observed a significantly higher number of cells undergoing anaphase with UFBs compared to control (Fig. 3F), indicating the presence of under-replicated CFSs (32). Lack of RPA staining on the anaphase bridges excluded the presence of extensive regions of ssDNA (Fig. 3F). The presence of under-replicated DNA in mitosis was confirmed by the characteristic accumulation of FANCD2 in symmetrical foci on the chromosome arms (Fig. 3G) and anticentromere antibody (ACA) staining revealed that the PICH-positive bridges caused by ICRF193-treatment were not associated with centromeres (Supplementary Fig. S3E).

p53 binding protein 1 (53BP1) is known to accumulate in foci on replication stress or incomplete replication. As previously demonstrated (16,33), 53BP1 nuclear foci decrease through S-phase progression in normal diploid cells (Supplementary Fig. S3F). Consistent with the delay in DNA replication progression, we found that 53BP1 accumulation in nuclear foci persists through S-phase upon Topo2a-inhibition and co-localises with γH2AX (Supplementary Fig. S3F) suggesting that 53BP1 is required to shield DNA lesions induced by ICRF193 during S phase. In addition, as in asynchronous cells, RPA immunostaining revealed the absence of single strand breaks (Supplementary Fig. S3G). It is noted that the sustained ICRF193-inhibition of Topo2a into G2, leads to loss the S-phase γH2AX staining (Fig. 2D; Supplementary Fig. S2G&H), indicative of resolution of these lesions during the arrest.

Collectively, these data indicate that Topo2a inhibition through S-phase affects completion of DNA replication generating regions of under-replicated DNA that can be tolerated, do not trigger the S-phase checkpoint, nor the Topo2a-dependent G2 arrest and can persist into mitosis. However, the bulk of DNA synthesis is completed before the ICRF193-mediated G2 arrest, as demonstrated by the DNA incorporation profile (Fig. 3H). By contrast, Topo1 inhibition by Camptothecin triggers an S-phase arrest that is not influenced by Topo2 inhibition, indicating the dominant role of Topo1 in S-phase DNA topological stress resolution (Fig. 3H).

### ALT-dependent cells have an alternative Chk1-mediated Topo2a-dependent G2 arrest

**Figure 4:**
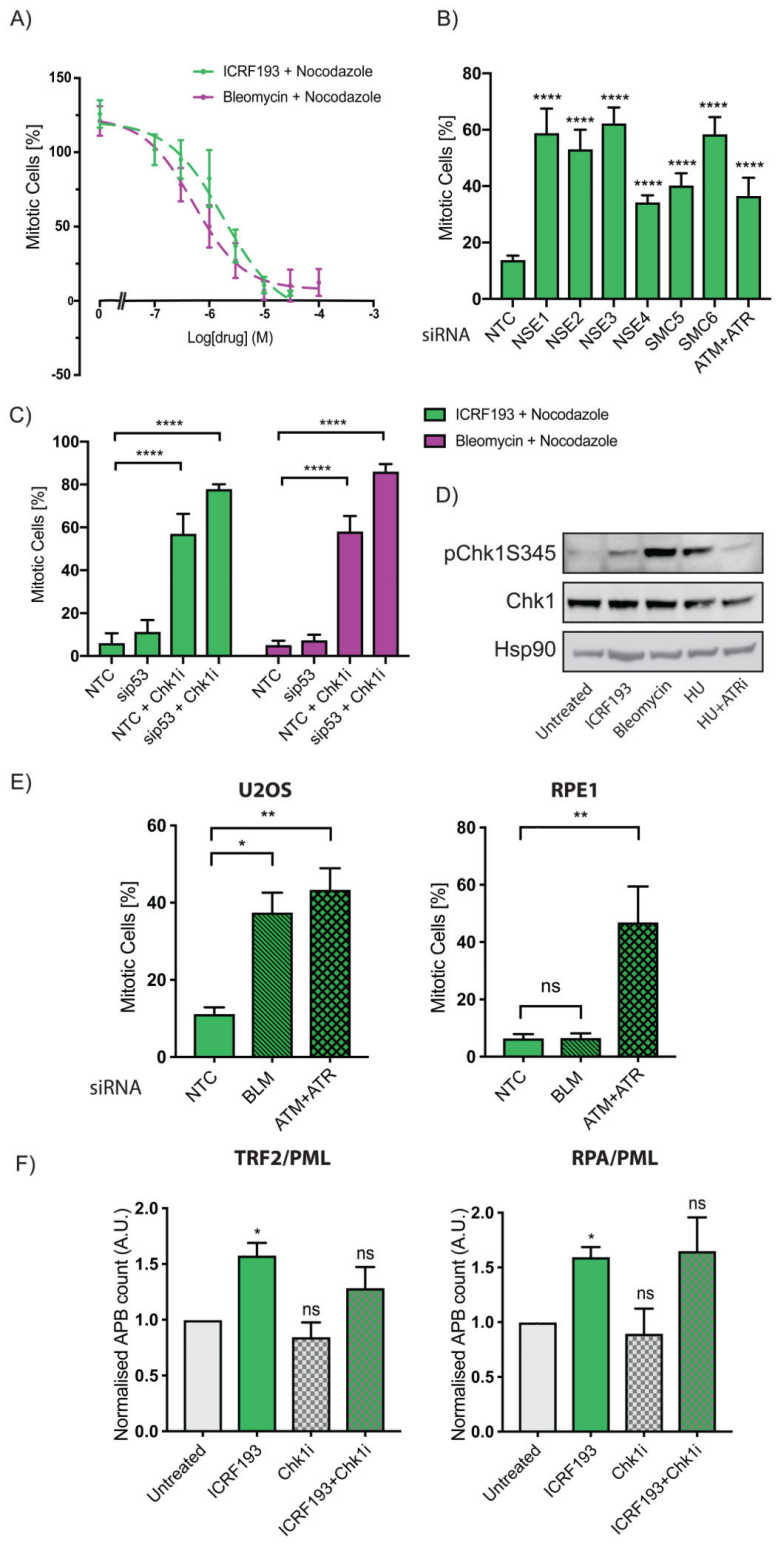
A) U2OS cells treated with ICRF193 or Bleomycin + Nocodazole for 24 h. Mitotic cells mean±SD are from a representative experiment with 8 technical replicates, n=3. B) Mitotic trap assay of U2OS cells transfected with the indicated siRNAs. Data are represented as mean±SD of a representative experiment with 6 technical replicates, n=3; analysis by oneway ANOVA. C) U2OS cells were transfected with control (NTC) or p53 siRNA (sip53), synchronised in G1/S, released for 10 h and treated for 16 h with ICRF193 or Bleomycin + Nocodazole and Chk1 inhibitor CCT244747 (Chk1i) where indicated. Mitotic index mean±SEM normalised to 16 h of nocodazole, n=3; analysis by two-way ANOVA. D) Western blots of U2OS cell extracts after 18 h of ICRF193, Bleomycin, ATR inhibitor (ATRi) or 2.5 mM hydroxyurea (HU) treatment. A representative experiment of n=3 is shown. E) Mitotic trap assay of U2OS and RPE1 cells transfected with control (NTC), siBLM or siATM+siATR and treated with ICRF193 + nocodazole for 18 h. Mitotic cells counts mean±SEM, n≥3; analysis one-way ANOVA. F) MATLAB-aided quantification of APBs (co-localisation of TRF2 or RPA) in U2OS cells after 18 h of treatment with ICRF193 and Chk1 inhibitor CCT244747 (Chk1i). Data normalised to untreated controls are mean±SEM, n=3; analysis t-test.

We observe an efficient G2 arrest in ICRF193-treated U2OS cells (Fig. 4A) (2), despite previous publications reporting they have compromised p53 signalling, lack a functional G1 DDR and their G2 DDR is reliant upon Chk1 (15,34). U2OS cells are p53 WT, but they have an active, truncated form of the phosphatase Wip1 (34), which we confirmed is present in our U2OS cell line (Supplementary Fig. S4A).

The ICRF193-mediated G2 arrest in U2OS cells is dependent upon known regulators of the Topo2a-dependent G2 arrest; ATM/ATR and all components of the SMC5/6 complex (Fig. 4B). Knockdown of p53 does not abrogate the ICRF193- or Bleomycin-induced G2 arrest in U2OS cells, consistent with the weakened p53 signalling previously described (Fig. 4C). However, we observed a dependence upon Chk1 with both ICRF193 and Bleomycin treatment in U2OS cells, starkly contrasting with normal, diploid cell lines (Fig. 4C; Supplementary Fig. S4B). Furthermore, upon ICRF193 treatment U2OS cells display Chk1 phosphorylation at Ser345 (Fig. 4D), in contrast to RPE1 cells (Fig. 2C).

Unlike other p53 defective cell lines studied here, U2OS cells are reliant upon ALT activity for telomere maintenance and immortality. Topo2a activity has been implicated previously in the ALT mechanism (8,10,12), furthermore ALT is reliant upon impaired p53 signalling and is reported to be dependent upon the SMC5/6 complex (35,36). To assess whether the ALT pathway correlates with this idiosyncratic arrest dependency, we investigated additional ALT cell lines, SAOS2 and GM847. Both initiated a partial arrest after ICRF193 treatment (Supplementary Fig. S4D), however this arrest, as in U2OS cells, was completely abrogated with the addition of a Chk1 inhibitor (Supplementary Fig. S4E). We hypothesise that Topo2a inhibition impacts telomere maintenance in ALT cells initiating an independent, distinct G2 arrest that while dependent upon ATM/ATR + SMC5/6 complex, relies upon Chk1 activity.

A recent study has described a BLM-dependent G2/M arrest in ALT cells, which occurred alongside an increase in telomere recombination intermediate dissolution and a subsequent increase in ALT phenotypes, including ALT-associated PML bodies (APBs) (37). Topo2a has previously been found in a complex with BLM and TRF2 in ALT cells and can enhance BLM activity in vitro (8,11). We confirmed that the ICRF193-mediated G2 arrest was dependent on BLM in U2OS cells and GM847 cells, but importantly this dependence was not observed in the normal, diploid cell line RPE1 (Fig. 4E; Supplementary Fig. S4F and G). In addition, ICRF193-treated U2OS cells reproduced the increase in APBs previously observed with hyper-ALT activity (Fig 4F; Supplementary Fig. S4H). These were also dependent on BLM presence and were not purely indicative of G2 arrested cells (Supplementary Fig. S4I&J). Chk1 activity is not required for the formation or maintenance of the hyper-ALT APBs, indicating a downstream role in the ICRF-induced arrest (Fig 4F; Supplementary Fig. S4H). Interestingly, we found the ICRF193-induced G2 arrest in ALT-dependent cells also led to G2 cellular senescence, but not as rapidly as observed in normal, diploid cells (Supplementary Fig. S4K&L).

### PKCε is engaged when the Topo2a-dependent G2 arrest is compromised by loss of the p53-p21 in hTERT positive cancer cells

**Figure 5:**
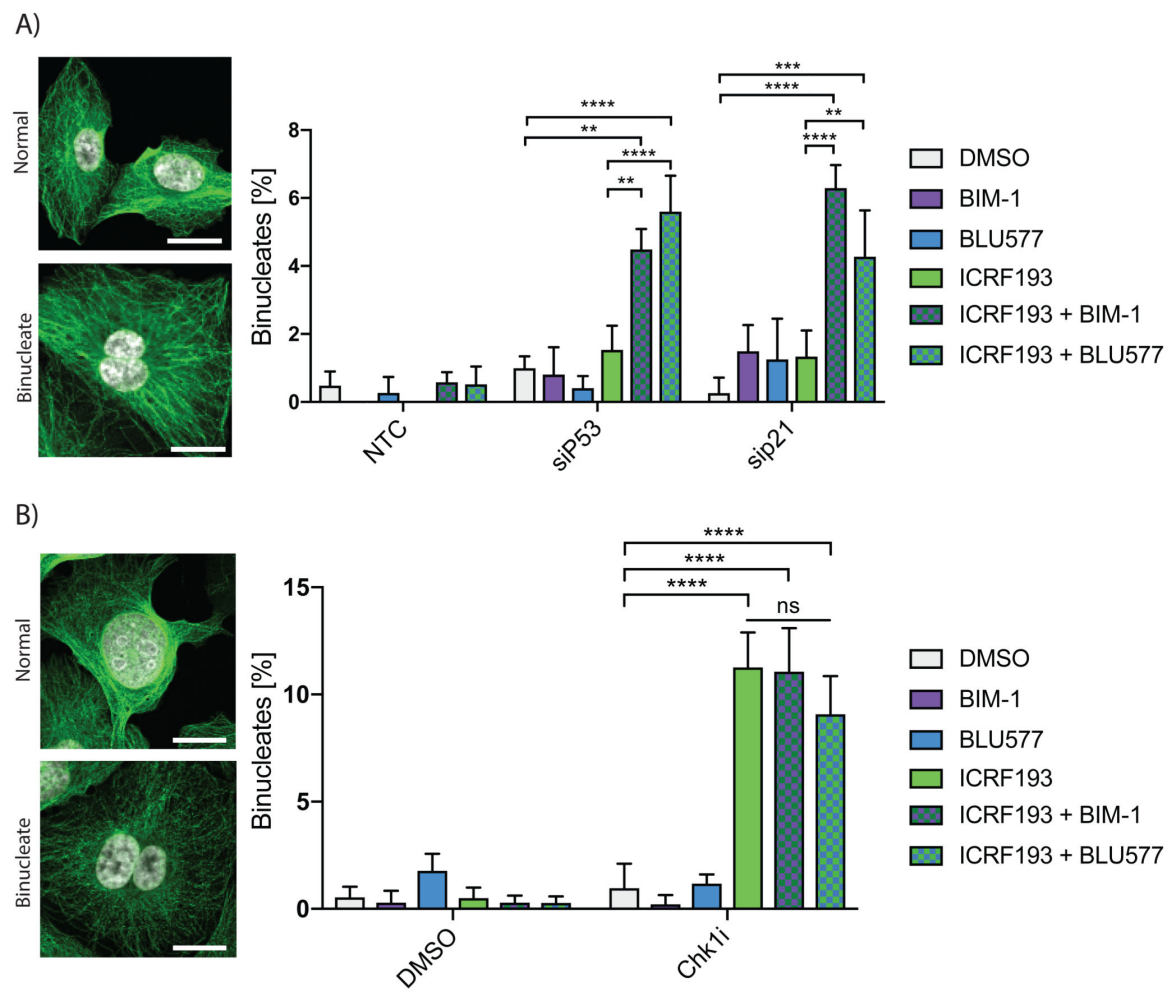
A,B) Representative confocal images show normal or binucleate cells with tubulin (green) and DAPI (white) staining. Scale bar = 20 μm. Data are represented as mean±SEM, ≥ 100 cells/experiment, n=3; analysis by two-way ANOVA. A) RPE1 cells were transfected with non-targeting control (NTC), siP53 OnTargetPlus or siP21 and were treated with DMSO, ICRF193, BLU577 or BIM-1 for 16 h. B) U2OS cells were synchronised with a single thymidine block, released for 10 h and then treated when in G2 with DMSO, Chk1 inhibitor CCT244747 (Chk1i), ICRF193, BLU577 or BIM-1 for 16 h.

We have previously identified three PKCε-regulated events that provide genome protection in cell lines with a compromised Topo2a-dependent G2 arrest (3). As p53 and p21 are essential for the Topo2a-dependent G2 arrest in normal, diploid cells, we tested whether experimental loss of p53 resulted in a dependence on PKCε for faithful chromosome segregation in RPE1 cells. The combination of ICRF193 and either of two structurally distinct inhibitors that can target PKCε, BLU577 and BIM-1, led to an increased number of binucleated cells only when either p53 or p21 were subjected to siRNA-mediated knockdown (Fig 5A).

To determine whether a by-pass in the ALT-associated ICRF193-induced arrest in U2OS cells also engendered reliance upon PKCε action, we scored the occurrence of failed division when Chk1 and PKCε were inhibited in combination. The loss of Chk1 activity resulted in an increase in binucleates, but this was not exacerbated by PKCε inhibition (Fig. 5B). Consistent with this, we observed an increase in DAPI positive bridges indicative of segregation errors with ICRF193 and Chk1 inhibitor treatment, which also was not exacerbated by PKCε inhibition (Supplementary Fig. S5A). Loss of the Topo2a-dependent arrest in ALT cells does not engage a PKCε dependent failsafe pathway.

### p53-PKCε display a genetic interdependence *in vivo*

To test the potential for PKCε and p53 to express a functional relationship *in vivo*, and in the absence of PKCε-selective drugs with suitable pharmacokinetic properties, we sought to test the impact of PKCε loss on tumours driven by p53 loss. Germline deletion of p53 in mice results in spontaneous tumorigenesis, mostly thymic CD4+CD8+ T-cell lymphomas (38). We observed that lack of PKCε in the p53-null tumour-prone model affected the age of tumour formation and survival, as Trp53^-/-^Prkce^-/-^ mice developed lymphoblastic lymphoma earlier than the Trp53^-/-^Prkce^+/+^mice (17.6 weeks vs 19.6 weeks, on average). However, PKCε germline deletion resulted in a less invasive phenotype, as Trp53^-/-^Prkce^-/-^ mice were found with enlarged thymi only. By contrast, Trp53^-/-^Prkce^+/+^ mice, developed thymic lymphomas involving other organs (Fig. 6A). Immunohistochemistry revealed elevated cell proliferation in the thymic samples from both genotypes, alongside caspase-3 activation (Supplementary Fig. 6A).

We assessed CNVs in these lymphomas and in accord with recent studies (39,40), the karyotypic landscape from the Trp53^-/-^Prkce^+/+^ tumours displayed aneuploidy for multiple chromosomes, including chromosome 4 and 5 gain and chromosome 13 loss (Fig. 6B), aneusomies identified also in human lymphoblastic lymphomas (41). Although the Trp53^-/-^ Prkce^-/-^ karyotype showed less whole-chromosome changes compared to the PKCε wild-type, the whole amplification of some specific chromosomes (such as chromosome 4, 5, 11, 14 and 15) and the reduction of chromosomes losses (Supplementary Fig. 6B&C), were consistently found among the samples analysed. In addition, we observed a higher number of intrachromosomal gains, especially on chromosome 12 and 4, and decreased intrachromosomal deletion events in Trp53^-/-^Prkce^-/-^, compared to the Prkce^+/+^ (Fig. 6C&D).

## Discussion

The study has determined that amongst the complex responses to Topo2 inhibition by the non-covalent catalytic inhibitor ICRF193, the cellular response is not governed by a singular signal relay, but by a context-dependent pattern of responses differentiated from the DDR. For S-phase, inhibition results in a delay to S-phase transition and an element of under-replication insufficient to trigger a G2 arrest, but if not resolved by the MiDAS pathway and carried through to anaphase results in PICH- and BLM-positive UFBs. In ALT cells we demonstrate the G2 arrest observed is dependent upon the SMC5/6 complex, ATM/ATR, BLM and Chk1. By contrast, the characteristic arrest observed in normal cells and hTERT immortalised cells is dependent upon the SMC5/6 complex, ATM/ATR, p53 and p21. Notably, the distinct relays also differentially engage the downstream failsafe pathway under PKCε control. Thus for cells by-passing the p53-dependent G2 arrest, there is an exacerbation of consequent division failure triggered by PKCε inhibition, which is not present in ALT cells. Finally we demonstrate that there is a genetic interdependence of p53 and PKCε *in vivo* in respect of the selection of specific chromosome aberrations associated with lymphomas, consistent with the functional relationship observed in the ex vivo models.

The requirement of p53 for a proficient Topo2a-dependent G2 arrest is in agreement with a previous observation in normal human fibroblasts (NHFs) (13), however we show that this behaviour is not a result of ICRF193-induced DNA damage or genotoxicity as previously concluded. However, our findings contrast with a study concluding that p53 is dispensable for an ICRF193-induced G2 arrest after 2 hours of treatment in NHFs (42), indicating that there may be a p53-independent acute phase delay that precedes a p53-dependent G2 arrest. Interestingly, data within this previous study supports this notion, where p53 loss in the NHF7 line results in an increased proportion of cells evading the drug-induced G2 delay after just 2 hours of ICRF193 treatment, which may reflect the acute window. We note that there are exceptions to this p53 arrest requirement. Conversely, cells that are p53 WT may lack a functional Topo2a-dependent G2 arrest response and be reliant upon PKCε-failsafe mechanisms due to deficiencies elsewhere in the signalling cascade.

The involvement of p53 rationalises why the cellular context in which the ICRF193-induced G2 arrest is studied is important. A substantial proportion of the literature on this arrest response has been conducted in p53 mutant or compromised cell lines, such as HeLa (14,43), where the dominant effect of ICRF193 is at the metaphase-to-anaphase transition (44). This could contribute to the differences in reported genes involved in the G2 arrest and the observed engagement of the DDR (in the absence of a robust arrest, ICRF193 will trigger aberrant divisions and their consequences). This issue is exemplified in the use HeLa cells to assess the levels of γH2AX as a readout for damage after ICRF193 treatment (15,45). The involvement of p53 can also account for the differential segregation errors observed upon treatment with ICRF193, with previous studies contrastingly demonstrating ICRF159, a structural relative of ICRF193, induced mostly centromeric UFBs when using cells immortalised with SV40 large T antigen that inhibits p53 function (46,47).

The identification of a distinct Topo2a-dependent G2 arrest in ALT cells clarifies the previous contradictory results demonstrating Chk1 involvement (15). The SMC5/6 complex has previously been shown to be essential for APB formation, ALT cell proliferation and maintenance (36) and previous studies have also shown Topo2a inhibition or knockdown causes an increase in telomere-damage induced foci (TIFs), an additional ALT cell marker (9,12). Therefore, we hypothesise that compromised Topo2a activity drives an increase in ALT-related properties with respect to APBs and a subsequent Chk1-mediated G2 arrest, elaborating upon the previously observed G2 arrest in response to an imbalance of dissolution at ALT telomeres. This Topo2a-dependent G2 arrest in ALT cells questions our previous conclusions, based upon the use of inducible U2OS cell lines to assess Topo2a mutants (2). It was concluded that SUMOylation of the novel site K1520 was not essential for a Topo2a-dependent G2 arrest but was involved in resolution. We cannot sustain this conclusion, but the observation does indicate that Topo2a SUMOylation at this site may also help facilitate resolution of ALT telomere intermediates. Using both RPE1 and primary patient-derived NSE2 mutant cells, we have previously shown that the E3 SUMO ligase activity is critical for both the G2 arrest and Topo2a-K1520 SUMOylation in response to ICRF193 (2). Therefore, it is likely that this SUMOylation site is required for a stringent Topo2a-dependent G2 arrest in normal, diploid cells, but this needs to be confirmed.

While the agents exploited here to block selectively PKCε activity are inadequate for assessing the impact of inhibition in the context of p53 defective tumours *in vivo*, we have provided evidence that there is an interdependence of p53 and PKCε in tumour development. Although this might be a tumour microenvironment consequence of PKCε loss, the finding that less aggressive tumours form when PKCε is absent and that there are consistent tumour autonomous changes in chromosome alterations, are consistent with tumour cell loss of the underlying PKCε-dependent genome protective pathway. This conclusion is bolstered by the observation that similar altered chromosomal changes were recently described in tumours isolated from mice where loss of p53 is combined with inactivation of the spindle assembly checkpoint protein Mad2 (40).

While it remains to be determined what is being monitored in the cells to trigger the non-ALT G2 arrest pathway, the characterisation of this important Topo2a-dependent G2 arrest offers a promising therapeutic opportunity. Given that p53 is the most frequently mutated gene in human cancer and where it isn't mutated its activity is typically compromised (48), many cancers will engage PKCε to support chromosome segregation. Therefore, targeting PKCε could prove beneficial therapeutically, exploiting the synthetic lethal behaviours in arrest-defective failsafe-reliant tumours. Such approaches are promising, as evident in the success of PARP inhibitors in BRCA mutant tumours (49). Furthermore, knowing that PKCε knock-out mice are viable adds the expectation that PKCε intervention would afford a good therapeutic index.

## Supplementary Material

Supplementary Figures and Tables

## Figures and Tables

**Figure 6:** A) Graph showing the age of death (weeks) of Trp53^-/-^Prkce^+/+^ and Trp53^-/-^Prkce^-/-^ mice. Mice showing enlarged thymus only (blue), thymus and spleen (pink) and thymus and metastasis (yellow). (B) Heat map showing DNA copy number changes in tumours from Trp53^-/-^ Prkce^+/+^ and Trp53^-/-^Prkce^-/-^. Values are presented for each chromosome (x-axis) from each mouse (y-axis) as a log2 ratio of amplification (red) or deletion (blue). (C,D) Graphs indicating single amplification or deletion events (y-axis) within each chromosome (x-axis) in the Trp53^-/-^Prkce^+/+^ (green) and Trp53^-/-^Prkce^-/-^ (orange) mice.

**Table 1:** p53 status of cells and their response to ICRF193.

Cell line	TP53 status	Details	Topo2a-dependent G2 arrest?	DNA Damage G2 arrest?
**hTERT-RPE1**	**WT**	**N/A**	**complete arrest**	**complete arrest**
**HFF**	**WT**	**N/A**	**complete arrest**	**complete arrest**
**BJ**	**WT**	**N/A**	**complete arrest**	**complete arrest**
**hTERT-BJ**	**WT**	**N/A**	**complete arrest**	**complete arrest**
**A549**	**WT**	**N/A**	**complete arrest**	**complete arrest**
**U2OS**	**WT**	**N/A**	**complete arrest**	**complete arrest**
**NCI H460**	**WT**	**N/A**	**complete arrest**	**complete arrest**
**HCC-4006**	**WT**	**N/A**	**partial arrest**	**complete arrest**
**NCI-H647**	**MUTANT**	**c.782+1G>T (splice donor mutation)**	**partial arrest**	**complete arrest**
**NCI-H2170**	**MUTANT**	**R158G**	**partial arrest**	**complete arrest**
**NCI-H1975**	**MUTANT**	**R273H**	**partial arrest**	**complete arrest**
**NCI H522**	**MUTANT**	**P191fs**	**partial arrest**	**complete arrest**
**NCI-H2228**	**MUTANT**	Q331*	**partial arrest**	**complete arrest**
**DLD-1**	**MUTANT**	**S241F**	**no arrest**	**complete arrest**
**ES2**	**MUTANT**	**S241F**	**no arrest**	**complete arrest**
**NCI-H727**	**MUTANT**	**InF Ins9c**	**no arrest**	**complete arrest**
**NCI-H520**	**MUTANT**	W146*	**no arrest**	**complete arrest**
**NCI-H1703**	**MUTANT**	**E285K**	**no arrest**	**complete arrest**
**NCI-H1792**	**MUTANT**	**c.672+1G>A (splice donor mutation)**	**no arrest**	**complete arrest**
**NCI-H1299**	**NULL**	**partial deletion**	**no arrest**	**complete arrest**
**HeLa**	**“NULL”**	**HPV18 E6 degrades protein**	**no arrest**	**complete arrest**

The published p53 status of the cells screened for their ability to arrest in response to ICRF193 is indicated. The nature of the induced arrest is colour coded as shown. The arrest of cells in response to bleomycin (DNA Damage G2 arrest) is also indicated (see Figure 1 and text for further details).

## Data Availability

The genomics data generated in this study are accessible at BioProject ID PRJNA807509.

## Statement of Significance

The identification of a requirement for p53 in stringent Topo2a-dependent G2 arrest and engagement of PKCε failsafe pathways in arrest-defective hTERT-positive cells provides a therapeutic opportunity to induce selective synthetic lethality.
