# Supplementary material for: Genome-protective topoisomerase 2a-dependent G2 arrest requires p53 in hTERT-positive cancer cells
Source: Cancer Res. Author manuscript; Available in PMC 2022 May 11. (PMC7612711; doi:10.1158/0008-5472.CAN-21-1785)
Supplement: Supplementary Figures and Tables [file EMS144799-supplement-Supplementary_Figures_and_Tables.pdf]

Table S1: A full list of biological reagents, chemicals, oligonucleotides and computational resources.

REAGENT	SOURCE	CATALOGUE NUMBER
**Antibodies**		
Rabbit polyclonal anti-53BP1 (H300)	Santa Cruz	#22760
Human polyclonal anti-Centromere protein antibody	Antibodies Inc	#15-234-0001
Goat polyclonal anti-ATM	Bethyl	#A300 135A
Mouse anti-phosphoATM (Ser1981)	Millipore	#05-740
Goat polyclonal anti-ATR	Santa Cruz	#sc-1887
Rabbit Phospho-(Ser/Thr) ATM/ATR Substrate Antibody	Cell Signalling Technology	#2851
Rabbit polyclonal anti-BLM	abcam	#ab2179
Mouse monoclonal anti-Chk1	Cell Signalling Technology	#2360
Rabbit polyclonal anti-Phospho-Chk1 (Ser345)	Cell Signalling Technology	#2431
Rabbit polyclonal anti-Chk2	Cell Signalling Technology	#2662
Rabbit polyclonal anti-Phospho-Chk2 (Thr68)	Cell Signalling Technology	#2661
Rabbit polyclonal anti-FANCD2	Novus	#NB100-182SS
Mouse monoclonal anti-GAPDH	Millipore	#MAB374
Mouse monoclonal anti-γH2AX	Millipore	#05-636
Rabbit Polyclonal anti-Histone H3	Cell Signalling Technology	#9715
Rabbit Polyclonal anti-Hsp90	Cell Signalling Technology	#4874
Mouse monoclonal anti-Lap2	BD Biosciences	#611000
Mouse monoclonal anti-MPM2-Cy5	Millipore	#16-220
Rabbit monoclonal anti-p21	Abcam	#ab109199
Mouse monoclonal anti-p53	Santa Cruz	#sc-126
Rabbit polyclonal anti-Phospho-p53 (Ser15)	Cell Signalling Technology	#9284
Rabbit polyclonal anti-Phospho-p53 (Ser20)	Cell Signalling Technology	#9287
Mouse monocolonal anti-PCNA (PC10)	Cell Signalling Technology	#2586
Rabbit polyclonal anti-PICH	Novus	#H00054821-D01P
Mouse monoclonal anti-PML	Abcam	#ab96051
Rabbit monoclonal anti-PML-Alexa Fluor555	Abcam	#ab217524
Mouse monoclonal [9H8] to RPA32/RPA2	Abcam	#ab2175
Rabbit polyclonal anti-SMC6	Abcam	#ab18039
Mouse monoclonal anti-TRF2	Millipore	#05-521
Mouse monoclonal anti-Tubulin	Sigma	#T5168
Mouse anti-Tubulin	In-house	N/A
Rabbit anti-Caspase 3	R&D Systems	#AF835
Rabbit anti-Ki67	Abcam	#ab15580
Mouse monoclonal anti-Wip1	Santa Cruz	#sc-376257
anti-mouse Alexa Fluor488	Invitrogen	#A11001
anti-mouse Alexa Fluor555	Invitrogen	#A21422
anti-mouse Alexa Fluor647	Invitrogen	#A21235
anti-rabbit Alexa Fluor488	Invitrogen	#A11008
anti-rabbit Alexa Fluor647	Invitrogen	#A31573
anti-rabbit HRP	Cell Signalling Technology	#7074
anti-mouse HRP	Cell Signalling Technology	#7076
**Chemicals**		
Aphidicolin	Sigma-Aldrich	#A0781
ATM Kinase Inhibitor	Merck	#118500
ATR Kinase Inhibitor	Merck	#504972
BIM1	Sigma-Aldrich	#203290
Bleomycin	Millipore	#203401
BLU577	Kindly provided by Dr Jon Roffey, Cancer Research Technology, UK	N/A
Camptothecin	Merck	#208925

Chk1 inhibitor CCT244747	Ref (22)	N/A
Chk1 inhibitor LY2603618	APExBIO	#A8638
Chk2 inhibitor CCT241533	Ref (23)	N/A
cOmplete™, EDTA-free Protease Inhibitor Cocktail	Roche	#4693159001
DAPI	Merck	#10236276001
EmbryoMax Nucleosides	Merck	#ES-008-D
Etoposide	Sigma	#E1383
Hydroxyurea	Sigma	H8627
ICRF193	Sigma	#I4659
Lullaby	OZ Biosciences	#LL73000
Luminata	Millipore	WBLUC0500
NuPAGE™ LDS Sample Buffer	Invitrogen	#NP0007
Nocodazole	Merck	#M1404
Phalloidin	Invitrogen	#A22283
PhosSTOP™	Roche	#4906845001
ProLong Diamond Antifade Mountant	Thermo Fisher	#P36965
Protein G Dynabeads	Invitrogen	#10004D
Propidium iodide	Sigma	#P4170
Ribonuclease A	Sigma	#R5125
RO3306	Tocris Bioscience	#4181
**Oligonucleotides**		
Non-targeting control pool	Dharmacon	#D-001810-10-05
siATM OTP pool	Dharmacon	#L-003201-00-0005
siATR OTP pool	Dharmacon	#L-003202-00-0005
siBLM OTP pool	Dharmacon	#L-007287-00-0005
siCDKN1A siGENOME Set of 4	Dharmacon	#MQ-003471-00-0002
siCHEK1 OTP Set of 4	Dharmacon	#LQ-003255-00-0002
siCHEK2 OTP Set of 4	Dharmacon	#LQ-003256-00-0002
siNDNL2 siGENOME Set of 4	Dharmacon	#MU-006402-00-0002
siNSMCE1 siGENOME Set of 4	Dharmacon	#MU-007157-01-002
siNSMCE2 siGENOME Set of 4	Dharmacon	#MU-018070-00-0002
siNSMCE4 OTP Set of 4	Dharmacon	#MU-017227-00-0002
siTP53 siGENOME Set of 4	Dharmacon	#MQ-003329-03-0002
siTP53 OTP Set of 4	Dharmacon	#LQ-003329-00-0002
siSMC5 siGENOME Set of 4	Dharmacon	#MU-014117-01-0002
siSMC6: GAGGGAAACGACAUUAUGA	Dharmacon	#D-018408-04-0010
siTOP2A siGENOME Set of 4	Dharmacon	#MU-004239-02-0002
**Organoid Culture Media and Additives**		
A83-01	Sigma	SML0788-5MG
B27	Thermo Fisher	#17504044
BME	R&D Systems	#35033-010-02
DMEM/F12	Gibco	#12634010
EGF	Stem Cell Technologies	#78006.1
Gastrin	Sigma	#G9145-1mg
Glutamax	Gibco	#35053361
HEPES	In House	N/A
N-acetylcysteine	Sigma	#A7250-5G
Nicotinamide	Sigma	#72345-50G
Noggin conditioned media	In House	N/A
Pen/Strep	Gibco	#15140-122
PGE2	Tocris Bioscience	#2296
R-spondin 1 conditioned media	In House	N/A
TrypLE Express	Thermo Fisher	#12604-013
SB202190	Sigma	#57067-5MG

Y-27632	Sigma	#SCM075
**Commercial Assays**		
Click-iT EdU Cell Proliferation Kit, Alexa Fluor 488 dye	Thermo Fisher	#C10337
**Software**		
MATLAB R2017B	MathWorks	https://uk.mathworks.com/products/matlab.html
FACSDiva	BD Biosciences	http://www.bdbiosciences.com/us/instruments/research/software/flow-cytometry-acquisition/bd-facsdiva-software/m/111112/features
FlowJo V.10.1	FlowJo, LLC	https://www.flowjo.com/solutions/flowjo
Prism 8	GraphPad	https://www.graphpad.com/scientific-software/prism/
Fiji	ImageJ	https://imagej.net/Fiji

Supplementary Table S2: A detailed list of cell lines used.

Cell line	Media (+10% FCS and 1% Pen/Strep)	Additives	% CO2	Gender	Cell line authentication	Source	Additional Information
**hTERT-RPE1**	DMEM	0.2mg/ml Hygromycin 1% NEAA*	10	Female	Yes	Cell Services STP Francis Crick Institute	
**HFF**	DMEM	-	10	Male	Yes	Cell Services STP Francis Crick Institute	
**BJ**	DMEM	-	10	Male	Yes	Yuneva Lab, Francis Crick Institute	
**hTERT-BJ**	DMEM	-	10	Male	Yes	Cell Services STP Francis Crick Institute	
**A549**	DMEM	-	10	Male	Yes	Cell Services STP Francis Crick Institute	
**U2OS**	DMEM	-	10	Female	Yes	Cell Services STP Francis Crick Institute	
**NCI H460**	RPMI	2mM Glutamine	5	Male	Yes	Cell Services STP Francis Crick Institute	
**HCC-4006**	RPMI	-	5	Male	Yes	Cell Services STP Francis Crick Institute	
**NCI H647**	RPMI	-	5	Male	Yes	Cell Services STP Francis Crick Institute	
**NCI H2170**	RPMI	-	5	Male	Yes	Cell Services STP Francis Crick Institute	
**NCI H1975**	RPMI	-	5	Female	Yes	Cell Services STP Francis Crick Institute	
**NCI H522**	RPMI	2mM Glutamine	5	Male	Yes	Cell Services STP Francis Crick Institute	
**NCI H2228**	RPMI	2mM Glutamine	5	Female	Yes	Cell Services STP Francis Crick Institute	
**DLD-1**	DMEM	-	10	Male	Yes	Cell Services STP Francis Crick Institute	
**ES2**	DMEM	-	10	Female	Yes	Cell Services STP Francis Crick Institute	
**NCI-H727**	RPMI	2mM Glutamine	5	Female	Yes	Cell Services STP Francis Crick Institute	
**NCI H520**	RPMI	1mM Sodium Pyruvate	5	Male	Yes	Cell Services STP Francis Crick Institute	
**NCI H1703**	RPMI	-	5	Male	Yes	Cell Services STP Francis Crick Institute	
**NCI H1792**	RPMI	-	5	Male	Yes	Cell Services STP Francis Crick Institute	
**HEK293**	DMEM		5	Female	Yes	Cell Services STP Francis Crick Institute	
**HEK293T**	DMEM		5	Female	Yes	Cell Services STP Francis Crick Institute	
**NCI H1299**	RPMI	-	5	Male	Yes	Cell Services STP Francis Crick Institute	
**HeLa**	DMEM	-	10	Female	Yes	Cell Services STP Francis Crick Institute	
**SAOS2**	DMEM	-	10	Female	Yes	Cell Services STP Francis Crick Institute	
**GM847**	DMEM	-	10	Male	Yes	Cell Services STP Francis Crick Institute	
**Patient fibroblasts NSE2 mutated** (p.Ser116Leufs*18/ p.Ala234Glufs*4)	DMEM, 15%FBS	-	5	Female	No	Semple Lab, University of Cambridge Metabolic Research Laboratories	Reference (30)
**Control fibroblasts**	DMEM, 15%FBS	-	5	Female	No	Semple Lab, University of Cambridge Metabolic Research Laboratories	Reference (30)
**Organoid cell line**	**Media**	**Additives**	**% CO2**	**Gender**	**Cell line authentication**	**Source**	**Additional Information**
**ORGANOID p53 WT**	DMEM/F12, 1% HEPES, 1% Glutamax, 1% Pen/Strep, 20% R-spondin1 conditioned media, 10% Noggin conditioned media	1.25 mM N-acetylcysteine, 10 mM nicotinamide, 500 nM A83-01, 10 mM SB202190, 50 ng/ml EGF, 10 mM PGE2, 10 nM Gastrin, 1x B27	5	Male	No	Cicarelli Lab, The Francis Crick Institute	
**ORGANOID p53 MUTANT T18 (R273H)**			5	Male	No	Cicarelli Lab, The Francis Crick Institute	Reported as pathogenic mutation in the DNA binding domain. Most frequently found in large intestine (https://cancer.sanger.ac.uk/cosmic/mutation/overview?id=96026314)
**ORGANOID p53 MUTANT T29 (T55fs)**			5	Female	No	Cicarelli Lab, The Francis Crick Institute	

*NEAA: non-essential amino acids

Figure S1.

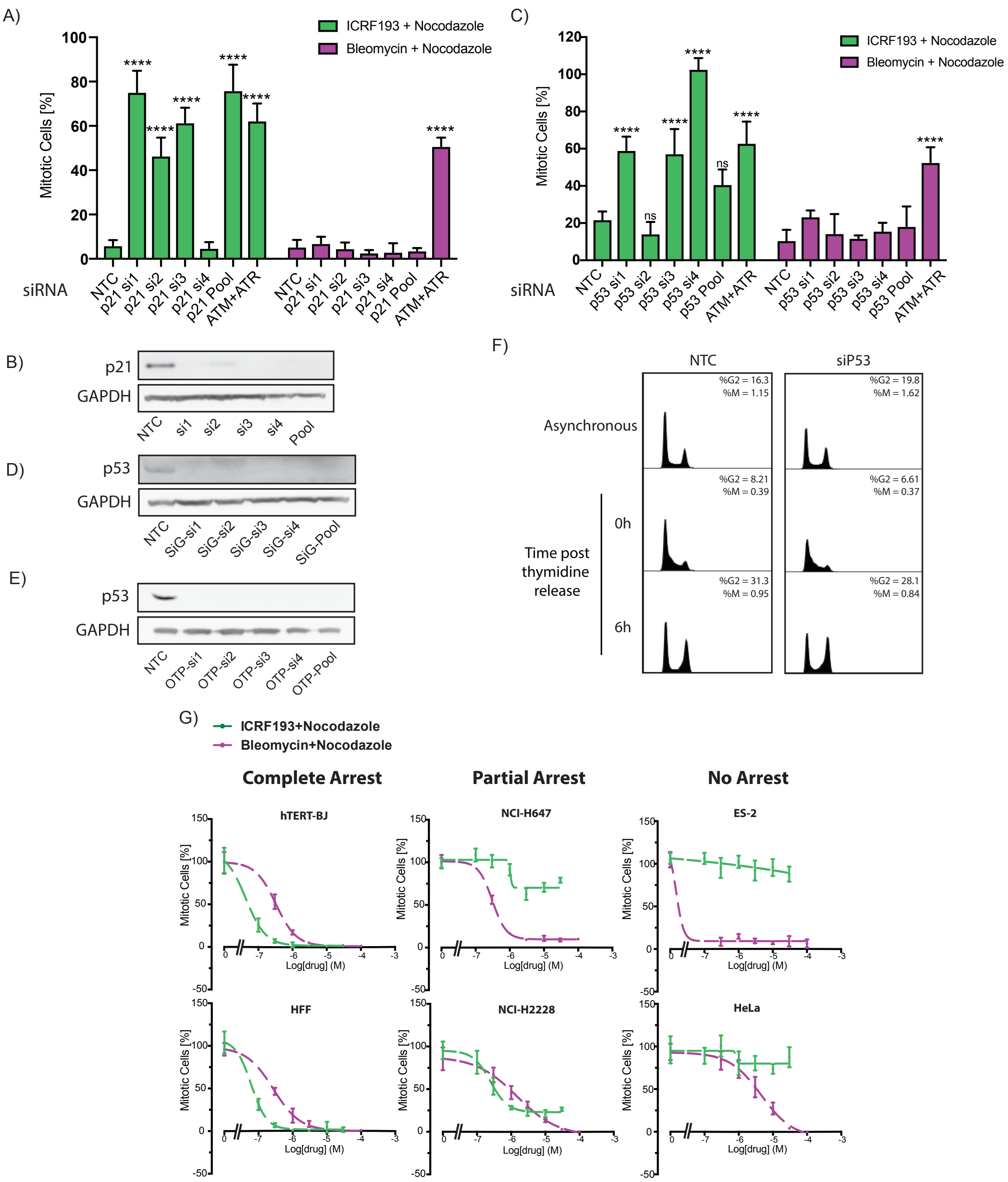

A) Mitotic trap assay of RPE1 cells transfected with p21 siRNA individually or as a pool alongside a non-targeting control (NTC) and positive control of siATM+siATR. Data are represented as mean ± S.D. of a representative experiment with 6 technical replicates, n=3. Analysis by a two-way ANOVA and compared to the NTC for each drug treatment. B) Western blots of RPE1 whole cell lysates with knockdown of individual p21 siRNAs and the combined pool, alongside a non-targeting control (NTC). A representative experiment of n=3 is shown. C) RPE1 cells transfected with siRNAs p53 SiGenome individually or as a pool alongside a non-targeting control (NTC) and positive control of siATM+siATR and treated and analysed as in (A). D-E) Western blots of RPE1 whole cell lysates with knockdown of (D)

p53 SiGenome (SiG) and (E) p53 OnTargetPlus (OTP) individual siRNAs and a combined pool, alongside a non-targeting control (NTC). Representative experiments of n=3 are shown. F) DNA profiles of RPE1 cells transfected with either non-targeting control (NTC) or siP53 OnTargetPlus Pool, showing no differences in cell synchrony or subsequent progression with p53 loss. The respective percentage of G2 and mitotic cells determined by MPM2 staining is displayed in the top right of each plot. G) Representative graphs for the indicated cell lines in response to increasing concentrations of ICRF193 or Bleomycin in combination with Nocodazole for 24 h. Mitotic cells were identified by MPM2 staining and automated IF analysis and were normalised to 1 μM of Nocodazole only. Data are represented as mean ± S.D. of a representative experiment with 8 technical replicates, n=2-3. The dashed non-linear regression line has been extrapolated to include the Nocodazole only condition, which is indicated by a concentration of 0 μM.

Figure S2.

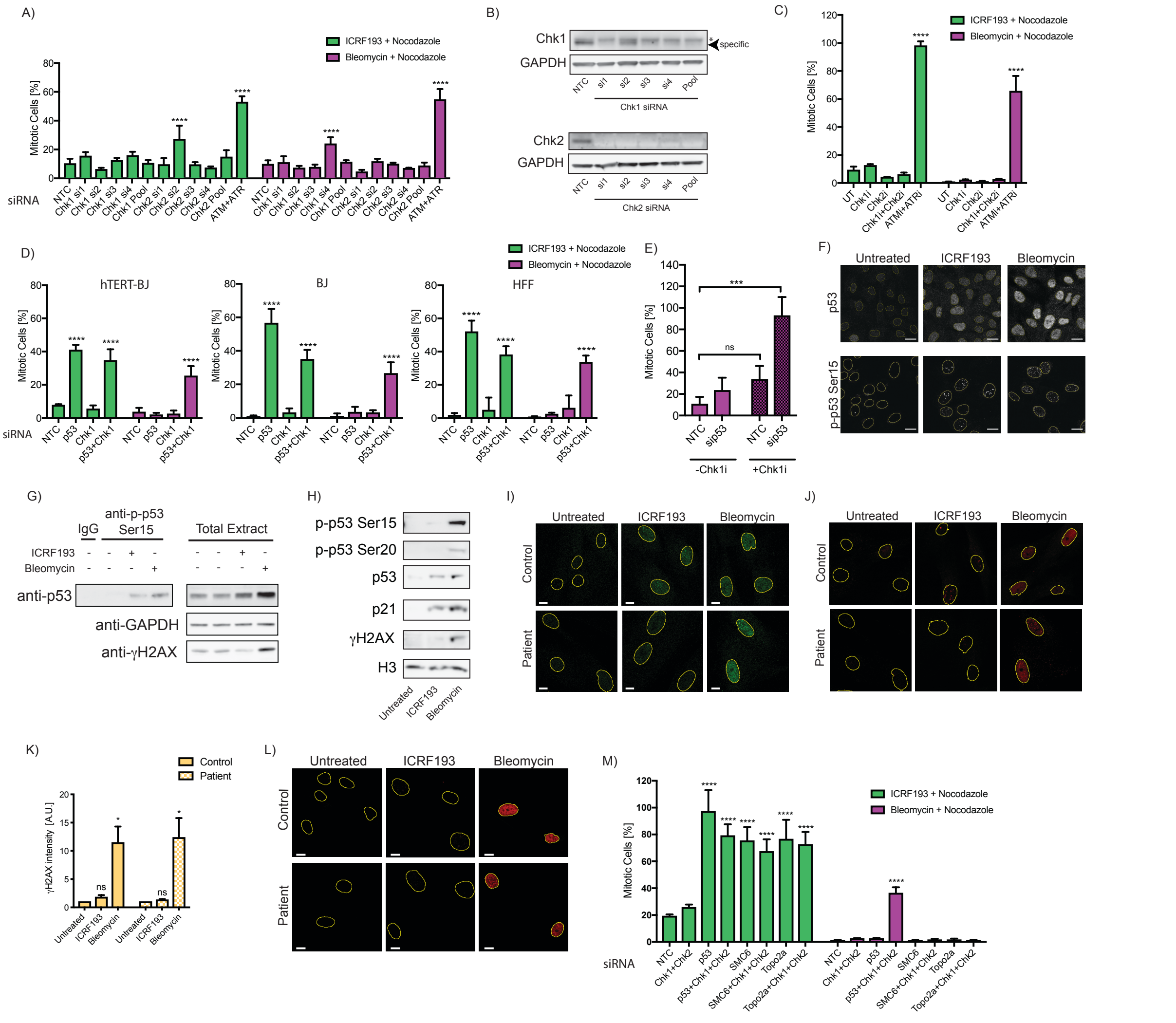

A) Mitotic trap assay of RPE1 cells transfected with siRNAs for Chk1 or Chk2 individually or as a pool alongside a non-targeting control (NTC) and positive control of siATM+siATR. Cells were treated with ICRF193 or Bleomycin in combination with nocodazole for 18 h. Data are represented as mean ± S.D. of a representative experiment with 6 technical replicates, n=3. Analysis by a two-way ANOVA and compared to the NTC for each condition. Chk1 si2 and Chk2 si4 were shown to be inconsistent and have off-target effects. B) Western blots of Chk1 and Chk2 knockdown efficiency in RPE1 cell lysates alongside Non-Targeting Control (NTC). Asterix denotes a non-specific band. A representative experiment of n=3 is shown. C) Mitotic trap assay of RPE1 cells that were either untreated (UT), treated with Chk1 inhibitor CCT244747 (Chk1i), Chk2 inhibitor CCT241533 (Chk2i) or ATM inhibitor ATR inhibitor (ATMi+ATRi). Data are presented as mean ± S.D. of a representative experiment with 6 technical replicates, n=3. Analysis by a two-way ANOVA and compared to the NTC for each condition. D) Mitotic trap assay of the indicated normal, diploid cell lines transfected with non-targeting control (NTC), siP53 OnTargetPlus and siChk1 as indicated. Data are presented as mean ± S.D. of a representative experiment with 6 technical replicates, n=3. Analysis by a

two-way ANOVA and compared to the NTC for each condition. E) HFF cells were transfected with non-targeting control (NTC) or p53 OnTargetPlus siRNA (sip53), synchronised with a double thymidine block, released for 8 h when in G2 and treated with Bleomycin + Nocodazole and with Chk1 inhibitor CCT244747 (Chk1i) where indicated for a further 16 h. The mitotic index was determined through flow cytometry, normalised to 16 h of nocodazole alone and represented as mean ± S.E.M., n=3. Analysis by a two-way ANOVA. F) Immunofluorescent staining of asynchrononous RPE1 for p53 or phosphorylation of p53-Ser15 (p-p53 Ser15) after 18 h of treatment with ICRF193 or Bleomycin as indicated. Nuclei are outlined in yellow as defined by DAPI staining. Scale bar = 10 μm. G) RPE1 cells were treated for 18 h with ICRF193 or Bleomycin and were subjected to immunoprecipitation with the phospho-p53 Ser15 (p-p53 Ser15) antibody. A representative western blot of n=3 is shown. H) Western blots of HFF nuclear extracts after treatment of cells for 18 h with ICRF193 or Bleomycin as indicated. A representative experiment of n=3 is shown. I,J) Immunofluorescent staining of asynchrononous Control and Patient fibroblast cells (Table S2) for (I) p53 or (J) phosphorylation of p53-Ser15 after 18 h of treatment with ICRF193 or Bleomycin as indicated. Nuclei are outlined in yellow as defined by DAPI staining. Scale bar = 10 μm. K) MATLAB-aided quantification of immunofluorescent expression of γH2AX of control or patient fibroblasts when treated with ICRF193 or Bleomycin for 24 hours as indicated. Expression levels are quantified per nucleus and are represented as mean ± S.E.M., n=4. Normalisation to the untreated control was used to account for biological replicates. Analysis was performed on treatment conditions using a one-sample t-test with a hypothetical value of 1. L) Immunofluorescent staining of asynchrononous Control and Patient fibroblast cells for γH2AX after 18 h of treatment with ICRF193 or Bleomycin as indicated. Nuclei are outlined in yellow as defined by DAPI staining. Scale bar = 10 μm. M) RPE1 cells were transfected with the indicated siRNAs alongside a non-targeting control (NTC) and treated and analysed as in (A).

<u>Figure S3</u>

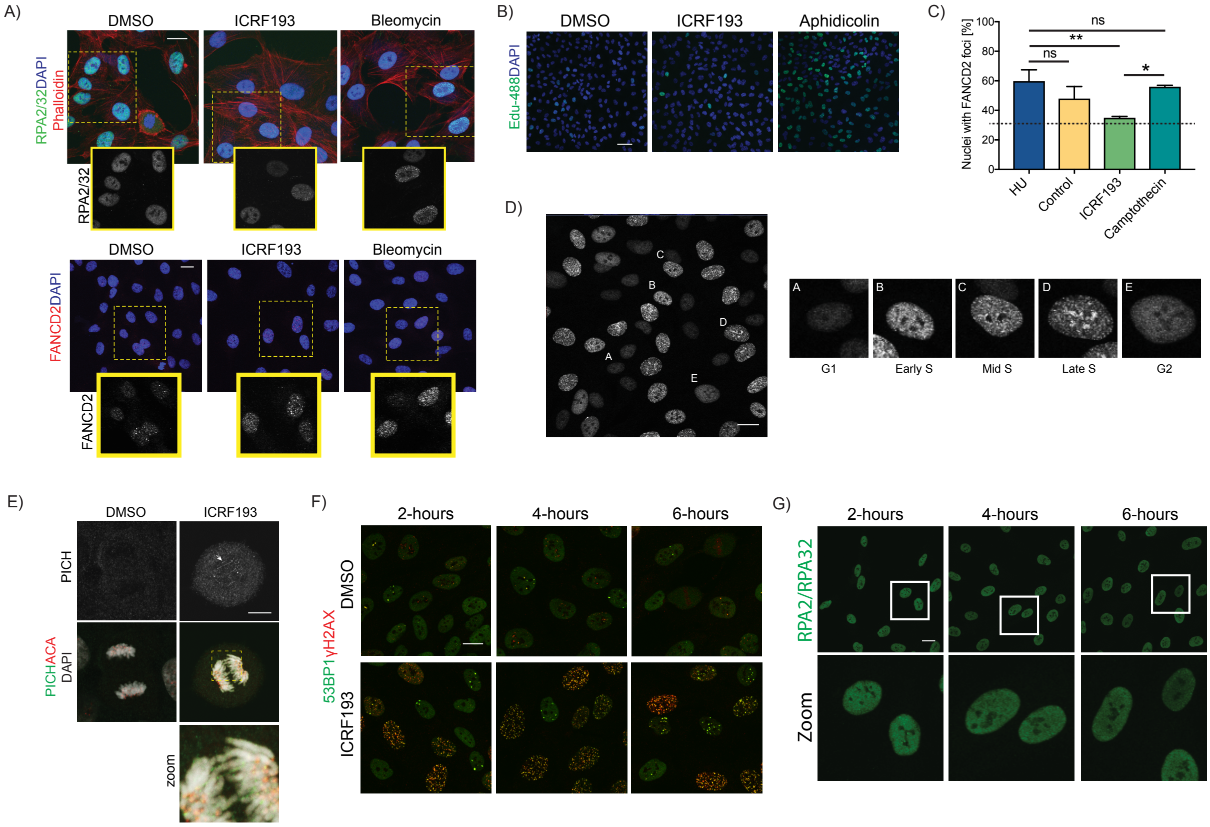

A) Representative immunofluorescence images of RPE1 cells treated with DMSO, ICRF193 and Bleomycin. Left: Cells are stained with anti-RPA2/32 (green), DAPI (blue) and Phalloidin (Red). Right: Cells are stained with FANCD2 (red) and DAPI (blue). Scale bars = 20 μm. B) Replicating RPE1 cells, treated with DMSO, ICRF193 or 0.5 μM Aphidicolin for 16 h, are detected combining EdU incorporation and Click-IT reaction. EdU in green and DNA in blue. Scale bar= 50 μm. C) RPE1 cells were synchronised in G1/S, released with Hydroxyurea (HU) for 45 minutes to induce replication stress and incubated in media containing the drugs indicated. Graphs show the quantification of FANCD2-positive foci per nuclei for each treatment. Dotted lines indicate the percentage of nuclei of asynchronous cells containing FANCD2 foci. Data are represented as mean ± S.E.M., n=3. Analysis by a one-way ANOVA and compared to HU for each drug treatment. D) Representative image of PCNA quantification from G1 to G2, as indicated in the zoom. Scale bar = 20 μm. E) Representative images of RPE1 cells synchronised in G1/S, treated with ICRF193 for 4 hours, washed to remove treatment and monitored into mitosis. Ultra-fine bridges in anaphase cells were detected staining for PICH (green) and centromeres using ACA (red). DNA was stained using DAPI. Scale bar = 10 μm. F) Representative images showing 53BP1 (green) and γH2AX (red) foci accumulation in RPE1 cells synchronised in G1/S and released with DMSO or ICRF193 for 2, 4 or 6 hours. Scale bar = 20 μm. G) Images of RPE1 cells stained with RPA2/RPA32 antibody (green), treated with ICRF193 for 2, 4 or 6 hours. Scale bar = 20 μm.

Figure S4.

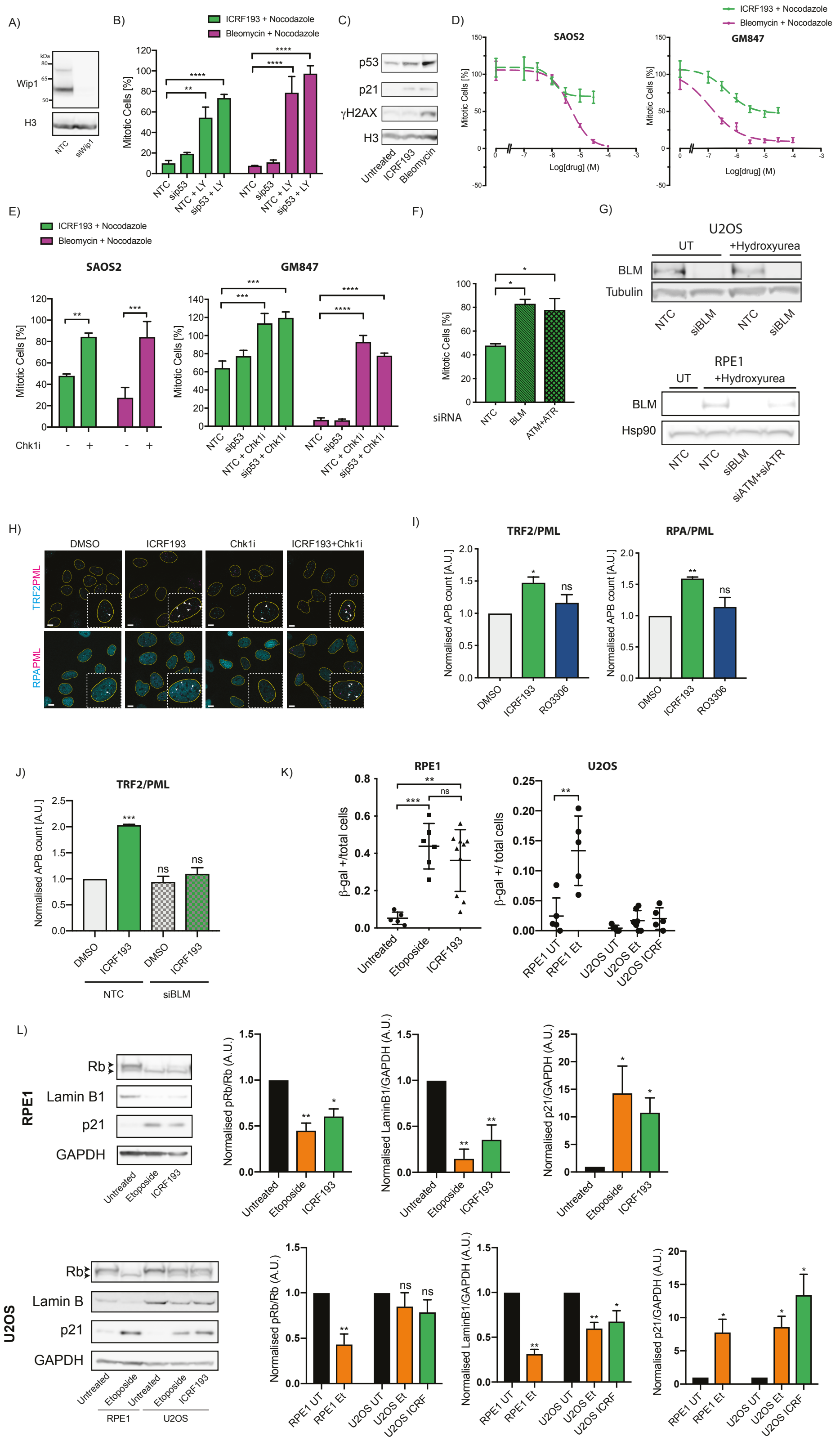

A)
Wip1
H3
NTC
siWip1
B)
ICRF193 + Nocodazole
Bleomycin + Nocodazole
Mitotic Cells [%]
NTC
sip53
NTC + LY
sip53 + LY
C)
p53
p21
γH2AX
H3
Untreated
ICRF193
Bleomycin
D)
SAOS2
GM847
Mitotic Cells [%]
Log[drug] (M)
E)
SAOS2
GM847
Chk1i
NTC + Chk1i
sip53 + Chk1i
F)
siRNA
BLM
ATM+ATR
G)
U2OS
UT
+Hydroxyurea
BLM
Tubulin
siBLM
RPE1
Hsp90
siATM+siATR
H)
DMSO
ICRF193
Chk1i
ICRF193+Chk1i
TRF2PML
RPAPML
I)
TRF2/PML
RPA/PML
Normalised APB count [A.U.]
RO3306
J)
TRF2/PML
NTC
siBLM
K)
RPE1
U2OS
β-gal +/total cells
Untreated
Etoposide
RPE1 UT
RPE1 Et
U2OS UT
U2OS Et
U2OS ICRF
L)
RPE1
Rb
Lamin B1
p21
GAPDH
Normalised pRb/Rb (A.U.)
Normalised LaminB1/GAPDH (A.U.)
Normalised p21/GAPDH (A.U.)
U2OS
Lamin B

A) Western blot of U2OS whole cell lysates confirming the Wip1 gain-of-function mutant in U2OS cells. A representative experiment of n=3 is shown. B) U2OS cells were transfected with non-targeting control (NTC) or p53 OnTargetPlus siRNA (sip53), synchronised with a double thymidine block, released for 10 h and then treated when in G2 with either ICRF193 or Bleomycin + Nocodazole and with 1 μM of Chk1 inhibitor LY2603618 (LY) where indicated for a further 16 h. The mitotic index was determined through flow cytometry, normalised to 16 h of 1 μM nocodazole alone and represented as mean ± S.E.M., n=3. Analysis by a two-way ANOVA. C) Western blot of U2OS whole cell lysates with either ICRF193 or Bleomycin as indicated. A representative experiment of n=3 is shown. D) ALT-dependent cell lines SAOS2 and GM847 were treated with increasing concentrations of ICRF193 or Bleomycin in combination with Nocodazole for 24 h. Mitotic cells were identified by MPM2 staining and automated IF analysis and were normalised to 1 μM of Nocodazole only. Data are presented as mean ± S.D. of a representative experiment with 8 technical replicates, n=3. The dashed non-linear regression line has been extrapolated to include the Nocodazole only condition, which is indicated by a concentration of 0 μM. E) Asynchronous SAOS2 cells were treated with either ICRF193 or Bleomycin + Nocodazole and with Chk1 inhibitor CCT244747 (Chk1i) where indicated for 24 h. GM847 cells were synchronised with a double thymidine block, released for 8 h and then treated in G2 with either ICRF193 or Bleomycin + Nocodazole and with Chk1 inhibitor CCT244747 (Chk1i) where indicated for 16 h. The mitotic index was determined through flow cytometry, normalised to 16 h of 1 μM nocodazole alone and represented as mean ± S.E.M., n=3. Statistical analysis was performed through a two-way ANOVA. F) Mitotic trap assay of GM847 cells transfected with non-targeting control (NTC), siBLM and siATM+siATR. Data represented as mean ± S.E.M., n=3. Analysis by a one-way ANOVA. G) Western blots of indicated whole cell lysates that were transfected with non-targeting control (NTC), siBLM or siATM+siATR for 72 h as indicated. Due to basal BLM levels being below the level for detection in RPE1 cells, 2 mM hydroxyurea (HU) was used in combination for 16 h. Representative experiments of n=3 are shown. H) Representative immunofluorescence images for APBs, TRF2 (blue)/PML (magenta) and RPA2/32 (blue)/PML (magenta), when U2OS cells were treated with ICRF193 and Chk1 inhibitor CCT244747 (Chk1i) as indicated. Nuclei are outlined in yellow. Arrowheads indicate co-localised foci. Scale bar = 10 μm. I,J) MATLAB-aided quantification of the number of APBs (determined by co-localisation of TRF2/PML and RPA/PML) in U2OS cells (I) with 18 h treatment with ICRF193 or 10 μM CDK1 inhibitor RO3306 and (J) transfected with non-targeting control (NTC), siBLM and with 18h ICRF193. Normalisation to the untreated control was used to account for biological replicates. Data are represented as mean ± S.E.M., n=3. Statistical analysis was performed on treatment conditions using a one-sample t-test with a hypothetical value of 1. K) Quantification of RPE1 and U2OS cells presenting β-galactosidase activity normalised to the total number of cells. Cells were treated with 12.5 μM Etoposide or 3 μM ICRF193 for 24 h as indicated. Data are presented as mean ± S.D. of a representative experiment with at least 5 fields of view encompassing at least 400 cells per condition, n=3. Statistical analysis was performed through a one-way ANOVA. L) Western blot analysis of whole cell lysates from RPE1 cells (top) and U2OS cells (bottom) treated with 12.5 μM Etoposide (Et) or 3 μM ICRF193 (ICRF) as indicated for 24 h. A representative experiment of n=3 is shown. Arrowheads indicate the phosphorylated (upper) and non-phosphorylated (lower) forms of Rb. Graphs denote the mean ± S.D. of 3 independent experiments where the expression level of proteins have been determined through densitometry. pRb expression levels (upper band) were normalised to Rb expression (lower

band), Lamin B1 and p21 expression were normalised to GAPDH expression. Normalisation to the untreated control was used to account for biological replicates. Statistical analysis was performed on treatment conditions using a one-sample t-test with a hypothetical value of 1.

Figure S5.

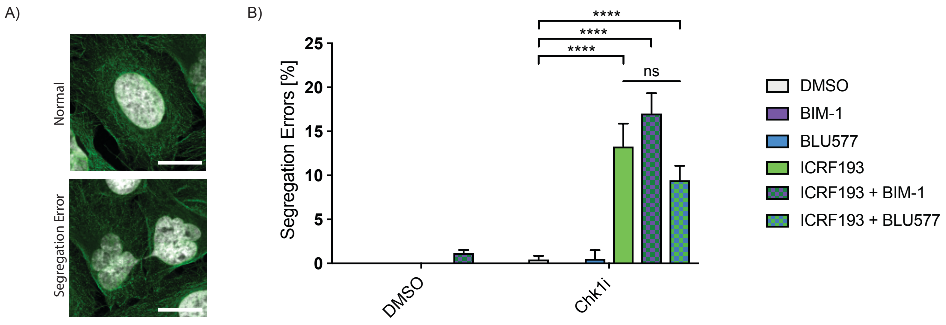

A) U2OS cells were synchronised with a single thymidine block, released for 10 h and then treated when in G2 with DMSO, Chk1 inhibitor CCT244747 (Chk1i), ICRF193, BLU577 or BIM-1 for 16 h as indicated before fixing and staining. Representative confocal images show nuclei that were normal or had DAPI positive bridges indicating segregation errors with tubulin (green) and DAPI (white) staining. Scale bar = 20 μm. B) Data as derived in A) are represented in the bar graph as mean ± S.E.M., where at least 100 cells were counted per experiment, n=3. Statistical analysis was performed through a two-way ANOVA.

Figure S6

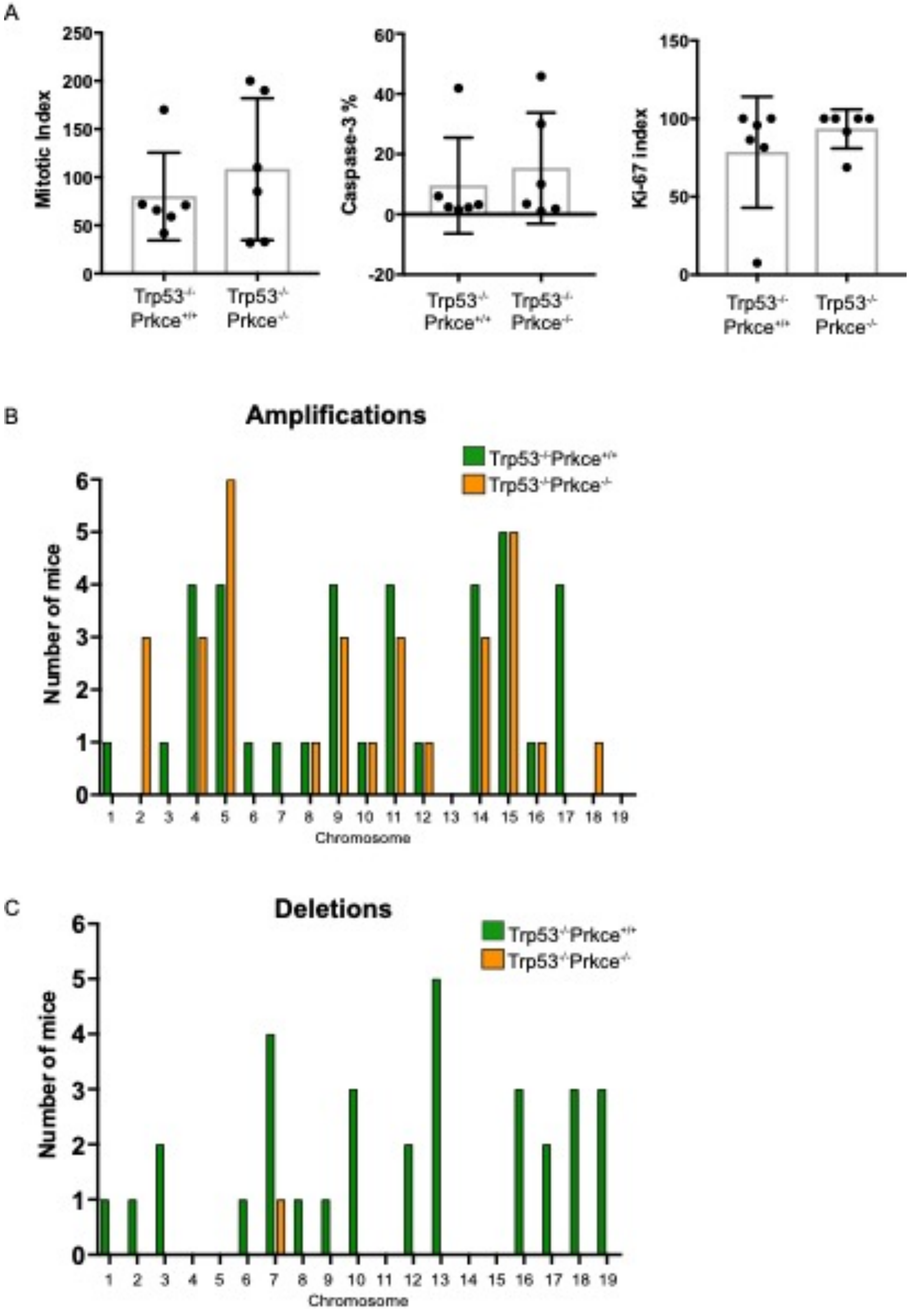

A) Graphs showing the quantification of mitotic cells (mitotic index), % of caspase-3 positive cells and quantification of Ki-67-positive cells (Ki-67 index) from the thymic tumours isolated from the Trp53$^{-/-}$Prkce$^{+/+}$ and Trp53$^{-/-}$Prkce$^{-/-}$ mice. B,C) Graphs show the number of Trp53$^{-/-}$

Prkce$^{+/+}$ (green) or Trp53$^{-/-}$Prkce$^{-/-}$ (orange) mice showing chromosome amplification (B) or deletion (C).
